# Endothelial cells in tumor microenvironment: insights and perspectives

**DOI:** 10.3389/fimmu.2024.1367875

**Published:** 2024-02-15

**Authors:** Patrizia Leone, Eleonora Malerba, Nicola Susca, Elvira Favoino, Federico Perosa, Giuliano Brunori, Marcella Prete, Vito Racanelli

**Affiliations:** ^1^ Internal Medicine Unit, Department of Interdisciplinary Medicine, Aldo Moro University of Bari, Bari, Italy; ^2^ Department of Precision and Regenerative Medicine and Ionian Area-(DiMePRe-J), Aldo Moro University of Bari, Bari, Italy; ^3^ Rheumatic and Systemic Autoimmune Diseases Unit, Department of Interdisciplinary Medicine, Aldo Moro University of Bari, Bari, Italy; ^4^ Centre for Medical Sciences, University of Trento and Nephrology and Dialysis Division, Santa Chiara Hospital, Provincial Health Care Agency (APSS), Trento, Italy; ^5^ Centre for Medical Sciences, University of Trento and Internal Medicine Division, Santa Chiara Hospital, Provincial Health Care Agency (APSS), Trento, Italy

**Keywords:** endothelial cells, tumor, microenvironment, T cells, tumor immune evasion

## Abstract

The tumor microenvironment is a highly complex and dynamic mixture of cell types, including tumor, immune and endothelial cells (ECs), soluble factors (cytokines, chemokines, and growth factors), blood vessels and extracellular matrix. Within this complex network, ECs are not only relevant for controlling blood fluidity and permeability, and orchestrating tumor angiogenesis but also for regulating the antitumor immune response. Lining the luminal side of vessels, ECs check the passage of molecules into the tumor compartment, regulate cellular transmigration, and interact with both circulating pathogens and innate and adaptive immune cells. Thus, they represent a first-line defense system that participates in immune responses. Tumor-associated ECs are involved in T cell priming, activation, and proliferation by acting as semi-professional antigen presenting cells. Thus, targeting ECs may assist in improving antitumor immune cell functions. Moreover, tumor-associated ECs contribute to the development at the tumor site of tertiary lymphoid structures, which have recently been associated with enhanced response to immune checkpoint inhibitors (ICI). When compared to normal ECs, tumor-associated ECs are abnormal in terms of phenotype, genetic expression profile, and functions. They are characterized by high proliferative potential and the ability to activate immunosuppressive mechanisms that support tumor progression and metastatic dissemination. A complete phenotypic and functional characterization of tumor-associated ECs could be helpful to clarify their complex role within the tumor microenvironment and to identify EC specific drug targets to improve cancer therapy. The emerging therapeutic strategies based on the combination of anti-angiogenic treatments with immunotherapy strategies, including ICI, CAR T cells and bispecific antibodies aim to impact both ECs and immune cells to block angiogenesis and at the same time to increase recruitment and activation of effector cells within the tumor.

## 1 Introduction

The vascular endothelium is a thin heterogeneous monolayer of very specialized cells, the endothelial cells (ECs), that line the luminal side of blood and lymphatic vessels, and form a barrier regulating the passage of substances, cells and pathogens from the blood to the tissues (1).

ECs differ in various organs and along the vascular tree in functions, cellular morphology (size, thickness and position of the nucleus), gene expression profile, cell surface properties, production of extracellular matrix, and expression of intercellular junctions (2, 3). The expression of the most common EC markers, such as CD31, CD34, and von Willebrand Factor (vWF) changes for distinct vessel types and different anatomic compartments of the same organ (4).

ECs are involved in numerous processes including the regulation of vascular hemodynamics, vascular permeability, coagulation, cell extravasation, fibrinolysis, inflammation and angiogenesis, and metabolic homeostasis (1, 3). As ECs are in direct contact with the blood flow, they can sense its hemodynamic changes and respond by the secretion of vasoactive substances. Moreover, ECs are characterized by great plasticity to adjust the vascular system to hemodynamic forces regulating the vascular tone. They instantaneously respond to flow stimuli with changes in cell morphology, electrochemical activities, and gene expression (5, 6). In addition, together with smooth muscle cells of the vessel wall, ECs regulate the blood flow to tissues by adjusting the degree of vascular relaxation/constriction. Blood flow regulation is essential also for the delivery and exchange of oxygen, fluids, nutrients and hormones that occur in the capillaries. The presence or absence of gaps on the endothelial monolayer, and the formation of extensive tight junctions among ECs and pinocytotic vesicles can regulate endothelium permeability and make the endothelium ‘continuous’ (e.g. gut-vascular barrier), ‘fenestrated’ (e.g. glomeruli in the kidney) or ‘discontinuous’ (e.g. liver sinusoids) (1).

Endothelial heterogeneity can arise also in response to stress or pathological conditions. The vascular endothelium is emerging as a dynamic organ able to modulate its environment and respond to external stresses, and as an important paracrine/endocrine organ that releases a wide variety of anti-inflammatory and pro-inflammatory vasoactive molecules, such as nitric oxide (NO), prostacyclin (PGI2), reactive oxygen species, endothelin-1 and tumor necrosis factor-alpha (TNF-α), growth factors, cytokines, metabolites of arachidonic acid and many peptides like endothelin, urotensin, adenosine, purines, and others (7). An imbalance in the synthesis and/or release of such mediators can be caused by stress events, inflammation, hypoxia, and cancer resulting in EC activation and dysfunctional disease-promoting endothelial phenotype (3). Once the activated stressor is removed, activated ECs can return to quiescent status through mechanisms that have not been clarified so far, but that involve EC intrinsic autophagy (8). When compared to normal ECs, tumor-associated ECs are abnormal in terms of phenotype, genetic expression profile, and functions. Tumor blood vessels appear irregular, fragile, and leaky, and the endothelial network seems chaotic with an anomalous blood flow (9–11). Evidence suggests that this abnormal vascular architecture contributes to tumor growth and metastasis (12). Tumor-associated ECs are characterized by great plasticity and the ability to trans-differentiate into mesenchymal cells through a process termed endothelial-to-mesenchymal transition (EndMT). In the context of cancer, EndMT is an important adaptive process of the tumor microenvironment that gives rise to cells with multipotent potential and promotes tumor proliferation, spreading, and resistance to chemotherapy (13).

Tumor-associated ECs exhibit stem cell-like origin thus being crucial for tumor neo-angiogenesis (14–16). Moreover, tumor development results in a hypoxic milieu, which stimulates the expression of hypoxia-inducible factor (HIF), pro-angiogenic chemokines and factors leading to a strong angiogenesis stimulation (17).

Tumor-associated ECs act also as gatekeepers for tumor-infiltrating immune cells and actively participate in the priming, activation, or downregulation of effector immune cells, thereby directly influencing antitumor immunity by mechanisms that remain partially understood. Furthermore, tumor-associated ECs can establish a link with immune checkpoint molecules and influence the response to antitumor therapies (18). They are involved in the formation of tertiary lymphoid structures (TLS) which contribute to boosting immune responses (19).This study aims to examine the critical role of ECs within the tumor microenvironment, with a particular focus on their relationship with immune cells and their impact on immunotherapy. A better understanding of tumor-associated EC functions could help identify tumor-associated EC specific drug targets to improve cancer therapy.

## 2 Endothelial cells in tumor microenvironment

Compared with ECs under physiological conditions (normal ECs), ECs in tumors display a markedly altered phenotype at the structural, molecular, and functional levels (**Table 1**). In general, disease promotes a loss of specialized capillary function and the acquisition of specific functions to support cancer cell survival. High tumor cell proliferation, tumor development and growth require the rapid formation of a complex vascular network which ensures sufficient oxygen and energy supply, and facilitates the removal of metabolic waste and carbon dioxide. Tumor growth results in a hypoxic microenvironment that cooperates with oncogenic processes to activate angiogenesis (20, 21). During tumor angiogenesis, ECs actively communicate with other angiogenesis driver cells, including pericytes, vascular smooth muscle cells, macrophages, skeletal muscle cells, and tumor cells through multiple mechanisms, such as cell-cell adhesion, formation of junctional complexes, secretion of paracrine cytokines and metabolites. Together these intercellular communications and the related cellular pathway activation form a very complex integrative signal network that drives angiogenesis and shapes endothelial phenotype (22). The coordinated execution and integration of such complex signaling programs is essential for physiological angiogenesis, while its dysregulation is critically linked to many major human diseases, including cancer. Angiogenesis is regulated by a large number of pro- and anti-angiogenic factors, whose levels dictate whether ECs will be in a quiescent or an angiogenic/activated state. When activators and inhibitors are in balance, the vasculature is quiescent and ECs are non-proliferative. During tumor development, pro-angiogenic factors and pro-angiogenic signaling pathways become dominant. Increased levels of HIF, vascular endothelial growth factor A (VEGFA), platelet-derived growth factor (PDGF), fibroblast growth factors (FGFs) or ANGPT2, as well as pro-angiogenic chemokines and receptors, lead to the “angiogenic switch”, the perpetual activation of ECs and the formation of new vessels from pre-existing vascular beds (23, 24). Through the binding to their receptors (VEGF receptor 2 (VEGFR2), PDGFR, TIE2), greatly upregulate on activated ECs, pro-angiogenic factors increase vascular permeability and promote EC proliferation, migration and assembly (23, 25).

**Table 1:** Main features of tumor endothelial cells (ECs).

**Morphology**	Irregular shape (ruffled margins and cytoplasmic projections extending outward and across the vessel lumen) Defective endothelial monolayer or multiple layers Intercellular gaps A discontinuous basement membrane An inconsistent coverage of smooth muscle and abnormal pericytes
**Phenotype**	High inter- and intratumoral heterogeneity Tip tumor ECs express CXCR4, PDGF, and ANGPT2 genes associated with EC migration, matrix remodeling and VEGF signaling Stalk ECs upregulate genes involved in vessel maturation and integrity as well as DII4-Notch signaling High expression of the c-Myc transcription factor High expression of CD34 (stem cell marker) and CD61 (angiogenesis), ALDH (drug resistance) High expression of ICAM-1 and VCAM-1 (immune cell homing), CXCL10 (chemotaxis) High expression of PD-L1, PD-L2 and IDO (T cell inhibition) Low expression of CD105, von Willebrand factor (vWF) Low expression of MHC-II, CD80 and CD86 (antigen presentation) Higher RNA content compared with normal ECs Highly proliferative phenotype Genomic and chromosomal instability Aneuploidy Abnormal multiple centrosomes Deletions Translocations Aberrant epigenetic profile Promoter hypermethylation and histone deacetylation of angiogenesis-suppressing genes DNA hypomethylation of genes involved in the control of EC growth and sprouting
**Function**	Line the interior surface of blood and lymphatic vessels Control vascular tone, blood fluidity, growth and homeostasis of the adjacent layer of smooth muscle cells Regulate coagulation, fibrinolysis, inflammation and angiogenesis Promote tumor proliferation, spreading, and resistance to chemotherapy Role of Gatekeeper for tumor infiltrating immune cells Role of Immuneregulator Form tertiary lymphoid structures (TLSs) Can transdifferentiate into mesenchymal cells (endothelial-to-mesenchymal transition, EndMT)

Moreover, pro-angiogenic factors can activate the genetic reprogramming of tumor-associated ECs supporting a highly proliferative phenotype with a great propensity for migration (26, 27). Hypoxia and reactive oxygen species (ROS) induce genetic and chromosomal instability through an elevated mutational frequency (28, 29). Fluorescence *in situ* hybridization (FISH) analysis showed that tumor-associated ECs are aneuploidy and have multiple karyotypic aberrations including abnormal multiple centrosomes, deletions and translocations (30). Furthermore, tumor-associated ECs display an aberrant epigenetic profile with silencing occurring in combination with promoter hypermethylation and histone deacetylation of angiogenesis-suppressing genes, and upregulation of genes involved in the control of EC growth and sprouting through DNA hypomethylation (31).

Morphologically tumor-associated ECs are characterized by irregular shape with ruffled margins and many cytoplasmic projections extending outward and across the vessel lumen. The intercellular connections are lost resulting in a defective endothelial monolayer or multiple layers with small and large intercellular gaps or holes, a discontinuous basement membrane, an inconsistent coverage of smooth muscle and abnormal pericytes (9, 10). This irregular and leaky architecture of tumor vessels facilitates the passage of fluid, blood, fibrin and tumor cells into the surrounding tissue promoting tumor metastasis (12). Furthermore, vessel leakiness increases the intratumoral interstitial pressure and reduces perfusion and blood flow causing tumor hypoxia and a metabolic EC switch to anaerobic glycolysis and acidosis (32).

Recent technological advances like single cell RNA sequencing (scRNAseq) were able to elucidate the wide inter- and intratumoral heterogeneity defining cellular subpopulations of tumor-associated ECs and providing functional information of the detected gene expression profiles. Tumor-associated ECs have a higher RNA content (two- to four-fold increase compared with normal EC) due to increased rates of transcription and high metabolic demands of nucleotide biosynthesis (purine and pyrimidine) or oxidative phosphorylation and glycolysis (15, 33). They exhibit elevated expression of the c-Myc transcription factor and its targets involved in tumor angiogenesis (15, 34). Compared with normal ECs, tumor-associated ECs display higher expression of the stem cell marker CD34 and the angiogenesis promoting markers CD61 (Integrin b3) and CD105 (Endoglin) (35), and lower or absent expression of von Willebrand factor (vWF) (36). Moreover, tip tumor-associated ECs express genes associated with EC migration, matrix remodeling and VEGF signaling such as CXCR4, PDGF, and ANGPT2, whereas stalk ECs upregulate genes involved in vessel maturation and integrity as well as DII4-Notch signaling (14, 37). Using scRNAseq in combination with orthogonal bulk multi-omics approaches, Goveia et al. have distinguished different subgroups of arterial, capillary and tip ECs expressing gene signatures related to basement-membrane breaching, vascular integrity, homeostasis, immune cell recruitment, and antigen presentation (14). In addition, they observed that specific EC phenotypes were differentially sensitive to anti-VEGF drugs and VEGFR tyrosine kinase inhibitors; tip and breach tumor-associated ECs were more sensitive to VEGF blockade than postcapillary vein and proliferating ECs, and capillary ECs were less sensitive to VEGFR tyrosine kinase inhibitors (14). Furthermore, the resistance to anti-VEGF therapy is due to the upregulation of alternative pro-angiogenic signals in tumor-associated ECs, such as genes involved in posttranscriptional collagen modification that could represent possible alternative angiogenic candidates (14).

However, selective targeting of different EC subpopulations *in vivo* is very difficult because of the limited number of truly unique surface molecules. Most of the reported EC targeting approaches are successfully restricted to organ-specific EC and not to a single EC subset (1).

Computational multi-pathway models that integrate multiple cell types and simultaneously simulate cell-cell communication have elucidated the individual molecular and cellular signaling components that function in concert to regulate angiogenesis. These models could also guide experimental studies to uncover new multilevel features of pathological angiogenesis and support the development of therapeutic strategies that target multiple pathways (22).

### 2.1 Stem-like origin of tumor-associated endothelial cells

The finding that tumor-associated ECs are highly proliferative and display abnormal gene expression profiles and chromosomal instability has suggested a stem cell-like origin. Stem cell-like ECs, also called endothelial progenitor cells (EPCs) with the capacity to self-renew and form blood vessels have been identified at the inner surface of preexisting blood vessels (38, 39). Recently, by scRNAseq several studies have identified ECs with a resident endothelial stem cell signature (14–16). However, the definition, cell lineage, and the specific mechanism by which EPCs differentiate into ECs are not yet known (40). A specific marker to identify EPCs is lacking given that potential typical markers such as CD34, CD31, VEGFR2 and CD133 are shared by vascular ECs and hematopoietic cells (myeloid cells and mesenchymal stem cells) (41). In glioblastoma has been found a CD133^+^ cancer stem-cell-like population which included a fraction of EPCs (42). Patel et al. have isolated mouse CD34^+^CD45^-^ vascular endothelial (VE)-cadherin^+^ EPCs with different expressions of CD31 and VEGFR2 based on the differentiation status which can develop entire blood vessels in wounds, inflamed skin, and tumors (43). Along with CD34 and CD133, tumor-associated ECs exhibit other stem cell markers including CD90, Sca-1, MDR1 ALP, and Oct-4, and form stem cell-like clusters with increased proliferative and invasive capacity and aldehyde dehydrogenase (ALDH) activity involved in resistance to therapy (26, 27, 44, 45).

Moreover, tumor cells can undergo vasculogenic mimicry in which they trans-differentiate into endothelial-like cells with the ability to develop matrix-rich vessel-like networks formed by vessels that do not arise from preexisting vessels (46). Interestingly, it was found that in glioblastoma and lymphoma, tumor cells can give rise to ECs. Indeed, they share the same somatic and cytogenetic mutations (42, 47).

The tumor can also produce molecules such as pleiotrophin and macrophage colony-stimulating factor (M-CSF) which guide the differentiation of monocytes and dendritic cells into endothelial-like cells (48, 49).

### 2.2 Immunoregulatory properties of tumor-associated endothelial cells

**Figure 1:**
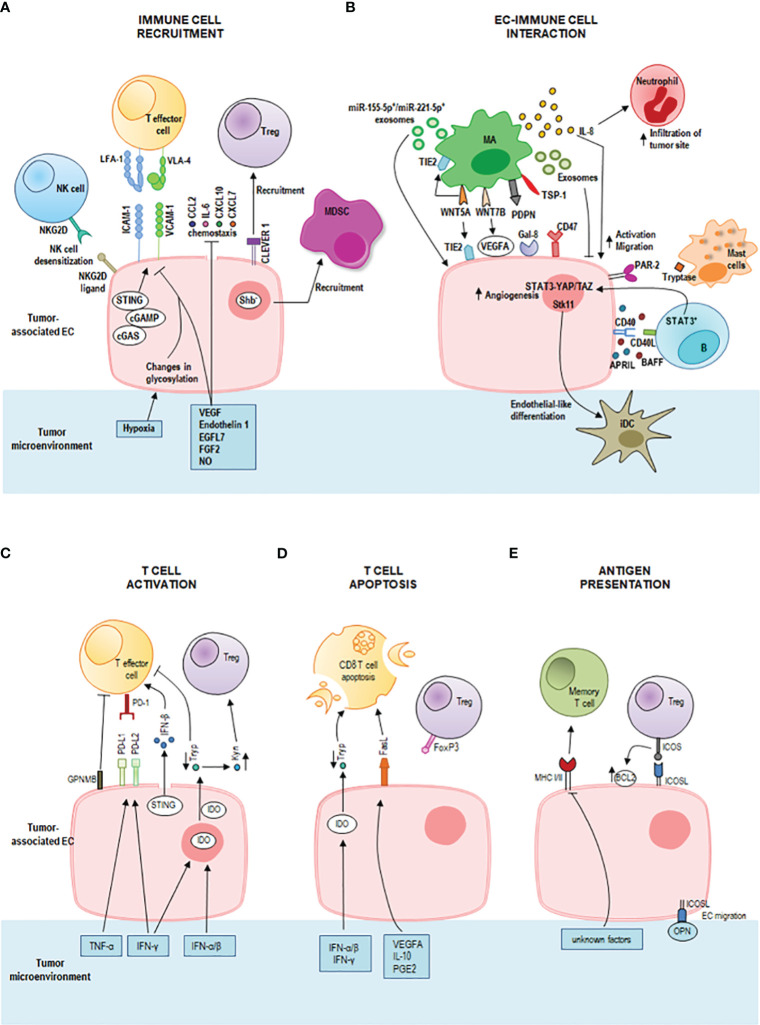
Immunoregulatory properties of tumor endothelial cells (ECs) in the tumor microenvironment. **(A)** Activated ECs recruit effector immune cells guiding their infiltration into the tumor microenvironment through a multi-staged adhesion process which includes binding of the integrins lymphocyte function-associated antigen-1 (LFA-1) and very late antigen-4 (VLA-4) on T cells to the respective ligands intercellular adhesion molecule-1 (ICAM-1) and vascular cell adhesion protein-1 (VCAM-1) on tumor-associated ECs. The tumor-deriving cytokines vascular endothelial growth factor (VEGF), endothelin-1 (ET1), EGF-like domain-containing protein 7 (EGFL7), and fibroblast growth factor 2 (FGF2) downregulate gene expression of adhesion molecules and chemoattractants (e.g., CCL2, IL6, CXCL10, and CXCL7) to inhibit immune cell infiltration. Also, nitric oxide (NO) in the tumor microenvironment inhibits immune cell recruitment. Tumor hypoxia results in changes in EC glycosylation which impair adhesion and cell-matrix interactions. Tumor-associated ECs represent a selective barrier that favors regulatory T (Treg) cell traffic by the common lymphatic endothelial and vascular endothelial receptor 1 (CLEVER 1), but represses effector T cells, dendritic cells, natural killer (NK) cells and neutrophil granulocytes promoting immunological tolerance. Tumor-associated ECs can cause desensitization of NK cells through the expression of NKG2D ligands that interact with the receptor NKG2D on NK cells. The intracellular cGAS-cGAMP-STING pathway can enhance adhesion molecules and promote T cell infiltration. Shb deprivation in tumor-associated ECs led to the recruitment of myeloid-derived suppressor cells (MDSCs). **(B)** IL-8 signaling promotes EC activation and the migration of tumor cells, ECs, and infiltrating neutrophils at the tumor site. Macrophage-derived WNT7B increases VEGFA expression on tumor-associated ECs. Macrophage-derived WNT5A stimulates EC proliferation and migration and upregulates TIE2 in macrophages (MAs) and ECs. Tumor-associated macrophages-derived exosomes suppress EC migration by the miR-146b-5p/TRAF6/NF-kB/MMP2 pathway, whereas M2 macrophage-derived exosomal miR-155-5p and miR-221-5p mediate macrophage-EC interactions. Podoplanin (PDPN) on tumor-associated MAs mediates the attachment of MAs to lymphatic ECs by interaction with Galectin-8 (Gal-8) and stimulates new lymph vessel formation and the migration of tumor cells through the lymphatic system. Tumor-associated MAs express thrombospondin-1 which interacts with CD47 receptor expressed by tumor cells and tumor-associated ECs. Downregulation of CD47 in tumor-associated ECs increases angiogenesis, vascular integrity and stability, VEGF-A and VEGFR2 expression. A bidirectional crosstalk between ECs and B cells is realized by CD40 on microvascular ECs and CD40L on tumor-associated B cells. This interaction increases the EC release of BAFF and APRIL which further enhance the expression of CD40L on B cells. Moreover, tumor-associated B cells stimulate ECs via the signal transducer and activator of transcription 3 (STAT3)-YAP/TAZ signaling. Tumor-associated ECs induce endothelial-like differentiation of immature dendritic cells (iDC) within the tumor microenvironment by the expression of serine/threonine kinase 11 (Stk11). Mast cells release tryptase which acts on the proteinase-activated receptor-2 (PAR-2) stimulating both vascular endothelial and tumor cell proliferation. **(C)** STING activates also T cells by inducing a strong IFN-β production. The pro-inflammatory tumor-derived cytokines IFN-γ and TNF-α increase the expression of the immune checkpoint ligands programmed death-ligand 1 (PD-L1) and programmed death-ligand 2 (PD-L2) on the tumor-associated EC surface. These ligands bind to the receptor programmed death 1 (PD-1) on T cells inhibiting T cell functions. ECs also induce tumor-infiltrating T cell exhaustion through the glycoprotein nonmetastatic melanoma protein B (GPNMB). Interferon (IFN)-γ, IFN-α and IFN-β upregulate indoleamine 2,3-dioxygenase (IDO) production by tumor-associated ECs promoting the metabolism of tryptophan (Tryp) to kynurenine (Kyn). Tryp depletion inhibits T cell proliferation, whereas Kyn accumulation promotes Treg differentiation and activation. **(D)** T cell apoptosis is triggered by IDO-mediated Tryp degradation and by upregulation of Fas ligand (FasL) on tumor-associated EC by tumor-derived VEGFA, interleukin (IL)-10 and prostaglandin E2 (PGE2). Tumor-associated ECs acquired the ability to kill effector CD8^+^ T cells but not Treg cells (due to expression of FoxP3). **(E)** Tumor-associated ECs can act as semi-professional antigen presenting cells. They can present processed antigens to memory T cells via class I and class II major histocompatibility complex (MHC) molecules, yet they do not express the co-stimulatory molecules CD80 and CD86 required for naïve T cell activation. They express the inducible co-stimulator ligand (ICOSL) which interacts with the receptor ICOS on Treg cells sustaining their activation and proliferation. ICOS/ICOSL interaction also increases the expression of the antiapoptotic protein Bcl-2. ICOSL can bind osteopontin (OPN) promoting EC migration.

Being endothelium a thin layer of cells that line the interior surface of blood vessels, ECs are in close contact with circulating innate and adaptive immune cells and mediate their interactions with tumor stroma. Tumor-associated ECs play a highly tuned role of sentinels and immune regulators by performing tissue-specific and vessel type-specific immunomodulatory functions including immune cell recruitment, activation and antigen presentation (**Figure 1**) (10, 50).

Whereas during immune homeostasis ECs allow immune cells to patrol by providing immune surveillance to the surrounding tissues, during inflammation, activated ECs recruit effector immune cells guiding their infiltration into the tumor microenvironment (51). The immune cell extravasation consists of three sequential steps, rolling, adhesion and transmigration (diapedesis) which are mediated by interactions between receptors and ligands on the endothelium and leukocytes, and involve vascular adhesion molecules and chemokines (**Figure 1A**). Initially, leukocytes establish transient selectin-mediated interactions with ECs, roll along the vessel wall, and are activated by chemokines presented on the endothelial surface. These chemokines by binding G protein-coupled receptor (GPCR) on leukocytes trigger an intracellular signaling cascade that induces the integrin-mediated firm adhesion of leukocytes by upregulation of integrin affinity for their respective ligands on ECs. As a last step, leukocytes pass across the vascular basement membrane and migrate along a chemotactic gradient toward the site of tissue damage (52, 53). Among the interactions between adhesion molecules and adhesion receptors, the binding of the integrins leukocyte function-associated antigen-1 (LFA-1) and very late activated antigen-4 (VLA-4) on T cells to the respective ligands intercellular adhesion molecule-1 (ICAM-1) and vascular cell adhesion molecule-1 (VCAM-1) on ECs critically regulates leukocyte adhesion and spreading (54).

During pathological processes, such as chronic inflammation and cancer, the decrease of expression of leukocyte adhesion molecules along with endothelium dysfunction and endothelial anergy (the lack of response to inflammatory activation) can strongly impair immune cell migration and tissue infiltration. Specifically, tumors secrete cytokines such as VEGF, endothelin 1, EGF-like domain-containing protein 7 (EGFL7), and fibroblast growth factor 2 (FGF2) which induce downregulation of endothelial expression of selectins, adhesion molecules, chemokines to inhibit leukocytes migration and generate a tumor immune-privileged microenvironment (10). Also, NO in the tumor microenvironment inhibits immune cell recruitment (55). Shb deprivation in tumor-associated ECs enhances the recruitment of myeloid-derived suppressor cells (MDSCs) that confer a suppressive immune response, thus promoting tumor metastasis (56) (**Figure 1A**).

Tumor-associated ECs can establish a selective barrier allowing specific T cell subsets to infiltrate the tumor site more effectively. They favor Treg cell traffic (57), but repress effector T cells, dendritic cells, NK cells and neutrophil granulocytes promoting immunological tolerance (52).

Upon inflammation, tumor-associated ECs can also enhance the expression of specific adhesion markers, such as the common lymphatic endothelial and vascular endothelial receptor 1 (CLEVER 1), to direct immune cells. CLEVER 1 is constitutively expressed on lymphatic EC, on sinusoidal EC in the liver and spleen, and on high endothelial venules (HEVs), and its expression can be induced on tumor-associated EC in blood vessels, where it favors selective transmigration of Treg cells and type II macrophages from the blood into the tumor (58–61).

Changes in EC glycosylation promoted by the tumor hypoxic milieu contribute to EC dysfunction impairing endothelial barrier function, adhesion, cell-matrix interactions and cell signaling (62) (**Figure 1A**). Moreover, tumor hypoxia induces the chronic release of pro-angiogenic factors resulting in EC anergy (63). Of note, EC anergy is a reversible process and may be used as a therapeutic strategy. Preclinical data show that blocking the aforementioned mechanisms in tumor ECs increases the antitumor immune response. For instance, anti Clever 1 antibody treatment reduces the recruitment of Treg into the tumor and decreases tumor progression *in vivo* (59). Anti-VEGF therapies may contribute to the reprogramming of the tumor microenvironment supporting EC activation and effector T cell recruitment (64).

Through the expression of NKG2D ligands, tumor-associated ECs can interact with NK cells causing desensitization of antitumor NK responses (65) (**Figure 1A**). Moreover, cytokines such as TNF-α and IL-1β activate ECs and induce the release of CCL2 and CCL7 that recruit NK cells and the expression of ICAM-1 and VCAM-1 that enable a stable contact between ECs and NK cells. These findings suggest that these molecules might be potential targets to enhance NK cell infiltration into solid tumors and to increase the antitumor efficacy of NK cell therapy (66).

Tumor-associated ECs can interact with tumor-associated macrophages (**Figure 1B**) enhancing the metastatic potential of tumor cells by increasing endothelial permeability which promotes circulating tumor cell adhesion and transmigration into the tissue (67). In epithelial ovarian cancer, tumor-associated macrophages stimulate EC function by IL-8 production (**Figure 1B**) (68). IL-8 signaling triggers angiogenic responses in ECs, promotes proliferation and survival of endothelial and tumor cells, and increases the migration of tumor cells, ECs, and infiltrating neutrophils at the tumor site. In addition, stress and drug-induced IL-8 signaling has been shown to confer chemotherapeutic resistance in cancer cells (69).

Moreover, tumor-associated macrophages express high levels of WNT family proteins, especially of WNT7B and WNT5A (**Figure 1B**). WNT7B promotes the angiogenic switch and vascular remodeling by increasing VEGFA expression on tumor-associated ECs (70). WNT5A stimulates EC proliferation and migration and upregulates TIE2 in macrophages and ECs (71). TIE2-expressing macrophages can mimic vascular structure through the expression of EC markers and the formation of capillary-like structures in response to VEGF, paving the way for vessel maturation with replacement by true ECs (71). TIE2 is also involved in the regulation of endothelial permeability (72). Tumor-associated macrophage-derived exosomes can suppress EC migration by the miR-146b-5p/TRAF6/NF-kB/MMP2 pathway, and epithelial ovarian cancer-derived exosomes can reverse this process (73). In addition, M2 macrophage-derived exosomal miR-155-5p and miR-221-5p are involved in macrophage-EC interactions. By suppressing E2F2 expression in ECs, they promote angiogenesis and support the development of pancreatic ductal adenocarcinoma (74, 75). In breast cancer, a tumor-associated macrophage subset expressing podoplanin (PDPN) is involved in the attachment of this macrophage subset to lymphatic ECs. PDPN interacts with Gal-8 on lymphatic ECs to promote new lymph vessel formation and the migration of tumor cells through the lymphatic system. Experimental removal of PDPN-expressing macrophages or inhibition of Gal-8 significantly reduces tumor metastasis in mouse breast cancer models (76). Tumor-associated macrophages express the signal-regulatory protein α (SIRPα) and the thrombospondin-1 which interact with the CD47 receptor, a don’t eat me signal, expressed by tumor cells and tumor-associated ECs. Interestingly, the downregulation of CD47 in tumor-associated ECs increases angiogenesis, vascular integrity and stability, VEGF-A and VEGFR2 expression and promotes tumor growth (77). Thus, although the treatment with antibodies against CD47 can induce antitumor immune responses by blocking the inhibitory CD47-SIRPα signaling in tumor cells, it may also potentially promote tumor progression by blocking CD47 signaling in ECs. Furthermore, blocking CD47 confers radioresistance to ECs *in vitro* and protects soft tissue, bone marrow, and tumor-associated leukocytes in irradiated mice (78).

In chronic lymphocytic leukemia, it has been observed that the constitutive release of soluble BAFF and APRIL increased upon engagement of CD40 on microvascular ECs by CD40L aberrantly expressed on tumor cells. Endothelial BAFF and APRIL further amplified chronic lymphocytic leukemia cell survival by enhancing the expression of leukemic CD40L suggesting a bidirectional crosstalk between ECs and B cells (**Figure 1B**) (79). Moreover, tumor-associated B cells stimulate ECs via the signal transducer and activator of transcription 3 (STAT3)-YAP/TAZ signaling promoting tumor angiogenesis (**Figure 1B**) (80, 81).

In esophageal squamous cell carcinoma, the concomitant presence of tumor cells expressing high levels of high-mobility group box 1 (HMGB1) and peritumoral regions with high density of proliferating B cells is an unfavorable prognostic factor. These B cells activate pro-angiogenic phenotypes and promote the growth of both ECs and tumor cells (82)).

Altogether these findings identify (STAT3)-YAP/TAZ signaling, proliferating B cells as well as HMGB1 signals as potential therapeutic targets for anti-angiogenesis therapy.

ECs can regulate dendritic cell differentiation by the expression of serine/threonine kinase 11 (Stk11) (**Figure 1B**). Deletion of Stk11 in mouse ECs strongly reduces mature DC numbers and increases spontaneous tumorigenesis (83). The activation of MAPK/ERK1/2 signaling stimulates immature DCs to undergo endothelial-like differentiation within the tumor microenvironment derived from the human esophageal squamous cell carcinoma cells and represents an alternative pathway of tumor angiogenesis (84, 85).

Mast cells can also modulate angiogenesis through the release of classical pro-angiogenic factors and nonclassical pro-angiogenic granule-associated mediators. One of the latter is the tryptase which is released by mast cells after c-Kit receptor activation. Tryptase acts on the proteinase-activated receptor-2 (PAR-2) stimulating both vascular endothelial and tumor cell proliferation (**Figure 1B**) (86).

ECs are also the main source of spontaneous IFN-β production in growing tumors, a molecule involved in the generation of spontaneous antitumor immune responses (87). The release of IFN-β upon recognition of intracellular foreign nucleic acids is driven by the cyclic GMP-AMP synthase (cGAS)-stimulator of interferon genes (STING) pathway, which has emerged as a critical link between innate and adaptive immunity (88) (**Figure 1C**). It has been demonstrated that intratumoral administration of exogenous cGAMP or STING agonist (cyclic diguanylate monophosphate; c-di-GMP) increases endothelial STING expression and IFN-β release and strongly boosts antitumor immunity leading to control of tumor growth in a mouse model of melanoma, breast cancer and glioma (87, 89–92). In murine glioblastoma models, treatment with STING agonists associated with biodegradable intracranial implants has demonstrated a profound shift in the tumor immune landscape, with massive intratumoral infiltration of innate immune cells, such as NK cells, and an increase in survival (89). This finding warrants further examination of STING agonists alone or in combination with other immunotherapies. The combination of cytotoxic cationic silica nanoparticles (CSiNPs) with STING agonists can activate tumor-infiltrating antigen-presenting cells resulting in increased expansion of antigen specific CD8^+^ T cells, and potent tumor growth inhibition in murine melanoma (93). Despite the very encouraging preclinical results, limitations to the use of CSiNPs-STING agonists in anticancer therapy lie in their chemical features.

Endothelial STING expression is also associated with normalizing of tumor blood vessels, increased adhesion molecule expression, enhanced T cell infiltration, and prolonged survival in human colon and breast cancer (94).

In addition, STING activation synergizes with VEGFR2 blockade and/or immune checkpoint inhibitors (ICI) leading to normalization of the tumor vasculature and the tumor microenvironment, significant upregulation of adhesion molecules (ICAM-1 and VCAM-1) on ECs, and complete regression of immunotherapy-resistant tumors (90, 94). Further examination of this combination strategy of STING-based immunotherapy is ongoing in clinical trials.

The pro-inflammatory cytokines IFN-γ and TNF-α enhance the expression of the immune checkpoint ligands programmed death-ligand 1 (PD-L1) and programmed death-ligand 2 (PD-L2) on the tumor-associated EC surface. These ligands bind to the receptor programmed death 1 (PD-1) on T cells inhibiting T cell functions (**Figure 1C**). Recently, Sakano et al. have demonstrated that hepatocellular carcinoma-associated ECs can induce tumor-infiltrating T cell exhaustion through the expression of the glycoprotein nonmetastatic melanoma protein B (GPNMB), suggesting that GPNMB might be a novel therapeutic target in hepatocellular carcinoma (95) (**Figure 1C**).

Upon type I and type II IFN stimulation, tumor-associated ECs can express high levels of the immunosuppressive enzyme indoleamine 2,3‐dioxygenase (IDO) which is associated with T cell apoptosis, inhibition of T cell proliferation and activation of Treg cells (96, 97) (**Figures 1C, D**). IDO inhibition has been shown to enhance the efficacy of ICI and represents a promising strategy for cancer therapy. The combination of ICI with IDO inhibitors yields a synergistic effect in the activation of antitumor immune responses, and clinical trials to evaluate their efficacy are ongoing (98, 99).

Tumor-derived VEGFA, IL-10 and prostaglandin E2 cooperatively induce upregulation of the death mediator Fas ligand (FasL) in ECs which gain the ability to kill effector CD8^+^ T cells but not Treg cells (due to expression of FoxP3) (100) (**Figure 1D**). Pharmacological inhibition of these factors results in lower FasL expression on ECs and higher CD8^+^ T cell infiltration by preventing effector T cell apoptosis (100).

In addition, tumor-associated ECs can act as semi-professional antigen presenting cells and can activate memory T cells. They express both class I and class II MHC molecules and low levels of the co-stimulatory molecules CD40, CD80 and CD86 required for naïve T cell activation. Tumor-associated ECs express the inducible co-stimulator ligand (ICOSL) which sustains activation and proliferation of the Treg population, and increases its suppressor function (101, 102) (**Figure 1E**).

Moreover, the crosstalk between Treg cells and ECs via ICOS/ICOSL interaction increases the expression of the antiapoptotic protein Bcl-2 on the endothelial surface and improves the sensitivity of B-lymphoma cells towards ABT-199, a potent Bcl-2 inhibitor (103). In addition, a recent study has demonstrated that ICOSL can bind osteopontin, and their interaction induces EC and tumor cell migration (104) (**Figure 1E**).

### 2.3 High endothelial venules and tertiary lymphoid structures

**Figure 2:**
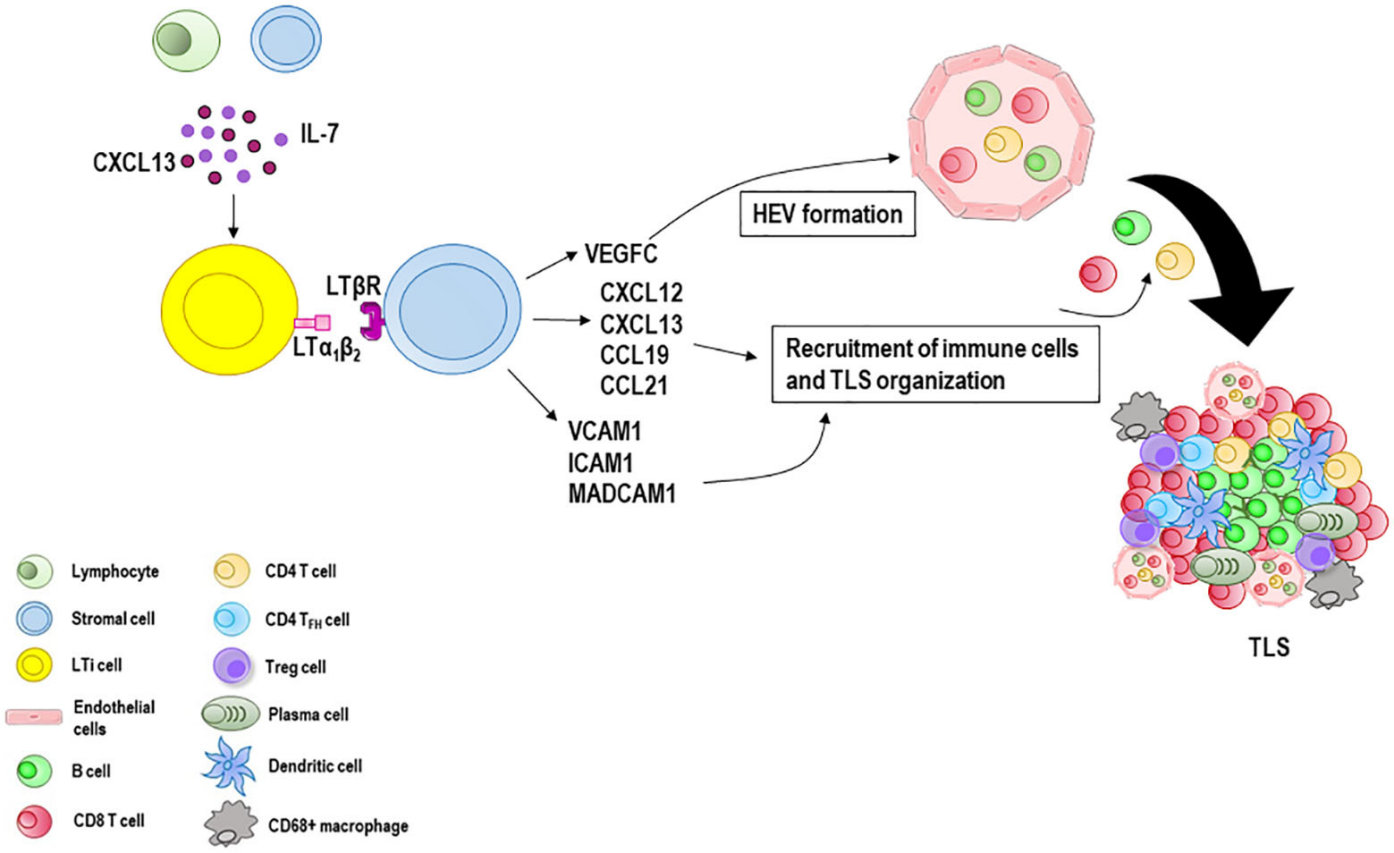
Tertiary lymphoid structures (TLS) formation and maturation. When immune responses fail to eradicate the triggering stimulus, the formation of TLS at sites with persistent inflammatory stimulation takes place. Lymphocytes and stromal cells release CXCL13 and IL-7 involved in the recruitment of lymphoid tissue inducer (LTi) cells which interact with local stromal cells via lymphotoxin-α1β2 (LTα1β2). Many immune cells such as B cells, M1 macrophages and Th17 cells can substitute LTi cells in the initiation of TLS genesis in various pathological conditions. Stromal cells secrete vascular endothelial growth factor C (VEGFC) which promotes the formation of the high endothelial venules (HEVs) and release adhesion molecules, such as vascular cell adhesion molecule 1 (VCAM1), intercellular adhesion molecule 1 (ICAM1) and mucosal addressin cell adhesion molecule 1 (MADCAM1), and various chemokines including CXCL12, CXCL13, CCL19 and CCL21. Together, these molecules recruit lymphocytes from HEVs and form a central B cell zone and a peripheral T cell zone enriched with CD8 T cells. CD4 T cells, CD4 T follicular helper (T_FH_) cells and Treg cells can also be present, as well as CD68^+^ macrophages and antibody-producing long-lived plasma cells.

If immune responses fail to eradicate the triggering stimulus, the formation of tertiary lymphoid structures (TLS) at sites with persistent inflammatory stimulation takes place. TLS are highly organized cellular aggregates resembling secondary lymphoid organs that develop in non-lymphoid tissues during chronic infectious diseases, autoimmune and inflammatory disorders, and cancers (19, 105). Like secondary lymphoid organs, their organization includes T and B cell compartmentalization, antigen presenting cells such as dendritic cells, stromal cells, conduits, and a highly organized vascular system of HEVs and lymphoid vessels (106–108). Although still partly unclear, TLS generation (**Figure 2**) is thought to be similar to that of secondary lymphoid organs considering the anatomical resemblance (109). Local production of CXCL13 and IL-7 by lymphocytes or stromal cells recruits lymphoid tissue inducer (LTi) cells (110) which interact with local stromal cells via lymphotoxin-α1β2 (LTα1β2) (111). Th17 cells, B cells, or M1-polarized macrophages can substitute LTi cells in the initiation of TLS genesis in various pathological conditions (112–115). Stromal cells release vascular endothelial growth factor C (VEGFC) which promotes the formation of HEVs and secrete adhesion molecules, such as VCAM1, ICAM1 and mucosal addressin cell adhesion molecule 1 (MADCAM1), and various chemokines, notably CXCL12, CXCL13, CC-chemokine ligand 19 (CCL19) and CCL21 (116). Together, these molecules work in concert to regulate the subsequent recruitment of immune cells and vascularization (117, 118). HEVs are blood vessels located in the TLS periphery and involved in immune cell recruitment and transmigration (119). Similarly to HEVs in lymph nodes, HEVs in TLS allow naïve and memory lymphocytes to pass from the bloodstream into the parenchyma of the tissue where they can interact with their cognate antigen. Moreover, HEV ECs can form pockets, a kind of “waiting areas”, in which lymphocytes reside for several minutes before entering the lymph node (120). Acting as lymphocyte portals, ECs lining HEVs express at their luminal surface high levels of ICAM-1, and depending on the organ and state of maturation, peripheral node addressin (PNAd) and/or mucosal addressin cell adhesion molecule (MAdCAM-1), ligands for the lymphocyte-homing receptor l-selectin (CD62L) that mediate the initial capture and rolling interactions of lymphocytes along the vessel wall (121, 122). Lymphocytes pass through the EC layer of the HEVs, crawl inside the perivascular channel and finally arrive at the lymph node parenchyma (123). Lymphocytes organize into T cell and B cell zones which compose the inner zone of TLS with a central B cell zone surrounded by a peripheral T cell-rich area. B cells undergo antibody class switching and somatic hypermutation becoming antibody-producing plasma cells (124). CD4^+^ and CD8^+^ T cells recognize peptide tumor antigens presented by antigen presenting cells and undergo activation, proliferation, and differentiation into CD4^+^ T follicular helper (T_FH_) cells and CD8^+^ effector memory cytotoxic cells which often represent the dominant subsets (124–126). CD4^+^ T helper 1 (T_H1_) cells and Treg cells can also be present (127), as well as CD68^+^ macrophages for clearance of apoptotic cells (128, 129). T_FH_ cells express CXCR5 and migrate at the interface between B and T cell zones, where they support the activation of antigen specific B cells, and the generation of memory B cells and antibody-producing long-lived plasma cells (130–132). A subset of regulatory IL-10^+^ B cells that coexists with Treg cells has also been described in human breast and ovarian cancers (133, 134).

Unlike secondary lymphoid organs, TLS lack a capsule and are exposed to local antigens, danger-associated molecular patterns and cytokines. They represent a niche in which immune cells interact with each other and with the surrounding microenvironment to generate or reactivate or potentiate the immune response (135). The presence of TLS has been related to robust immune reactions to clear infections (136), or to severe tissue destruction in autoimmune diseases (137). In tumors, TLS may exert either pro- or anti-tumor activities and their presence has been associated with variable outcomes. Important parameters for the impact of TLS on tumor control are the number, composition, and location. A high number of TLS within tumor microenvironment correlates with increased infiltration of adaptive immune cells and a better prognosis in ovarian carcinoma (138), non-small-cell lung carcinoma (NSCLC) (139), breast (140), renal cell, pancreatic (141), and hepatocellular cancers (142), gastrointestinal tumors (143) and melanoma (125, 144).

The proportion of mature dendritic cells in TLS able to activate effector T cells is strongly associated with better outcome and long survival in NSCLC and rectal cancers (145, 146). High proportions of Treg cells within TLS have been linked to worse outcomes in several types of cancer, including ovarian, breast and hepatocellular cancer (138, 147, 148), and in a mouse lung adenocarcinoma model (149).

The location of TLS is also crucial. The presence of TLS close to tumor beds is associated with favorable patient prognosis. In contrast, their location at a distance from tumor beds, outside the invasive margin, correlates with poor outcome (150).

Emerging evidence associates the presence of preexisting TLS with better response to immune checkpoint-blocking therapies in melanoma (144, 151), sarcoma (152, 153), NSCLC (153–155), renal cell carcinoma (151, 153, 156) and urothelial cancer (153, 157). The mechanism remains still unclear, but it could involve B cells and their interaction with T cells (151). TLS are sites for maturation, selection, clonal amplification, isotype switching of B cells, and generation of antibody-producing plasma cells which contribute to the antitumor response. Moreover, B cells can release an array of cytokines, through which they activate and recruit other immune effector cells, and they may act as antigen presenting cells, driving tumor specific T cell activation and expansion (151). However, pro-antitumor activities have also been described for B cells due to the existence of a heterogeneous population including regulatory B cells with immunosuppressive phenotype (133, 134, 158–160). A better understanding of these specific TLS mediators could optimize treatments aimed at enhancing TLS-dependent antitumor immunity safely and effectively. Recently, antitumor drugs, chemokines, cytokines, agonistic antibodies, and engineered dendritic cells expressing the T_H1_ cell transcription factor T-bet or CCL21 have been used in combination with traditional antitumor treatments to promote TLS formation, with good results. These agents, in combination, can turn cold immune-suppressed tumors into hot immunogenic tumors, promoting tumor infiltration by effector T cells and increasing the recruitment of immune cells that participate in TLS development (135). In a mouse model of breast and pancreatic tumors, the combined anti-VEGFR2 and anti-PDL1 therapy overcame T cell exhaustion phenotype and increased antitumor immune response stimulating TLS neogenesis (161). In a cohort of 59 patients with pancreatic ductal adenocarcinoma, an intradermal vaccine with an irradiated allogeneic granulocyte colony-stimulating factor (GM-CSF) in combination with cyclophosphamide increased TLS development (162). Furthermore, in patients with cervical neoplasia, enhanced TLS formation and maturation could be observed in regressing lesions after human papillomavirus vaccination (163).

### 2.4 Tumor-associated endothelial cells and lymphatic vessels

The development of lymphatic vessels and their function are regulated by the VEGFC/VEGFR3 signaling (164). Activation of VEGFC/VEGFR3 signaling in lymphatic endothelial cells (LECs) increases the proliferation of LECs and the formation of lymphatic vessels, leading to the increase of lymphatic metastasis of tumor cells (165). Overexpression of VEGFR3 and its ligand VEGFC has been found in several tumors including colorectal cancer, endometrial, prostate cancer, ovarian carcinoma and breast cancer, and is associated with increased formation of lymphatic vessels, lymph node and distant metastases, as well as a poor prognosis (166–170).

Additionally, the VEGFC/VEGFR3 signaling may also modulate antitumor immune response promoting immune tolerance. VEGFC was found to protect tumors against preexisting antitumor immunity in a mouse melanoma model. In these mice, VEGFC activated LECs were able to cross-present tumor antigens leading to dysfunctional activation of tumor-specific CD8^+^ T cells (171). Moreover, in patients with acute myeloid leukemia, low-functioning NK cells exhibited great levels of VEGFR3 which promotes chemotherapy resistance (172). In lung adenocarcinoma, tumor-associated macrophages induced the expression of VEGFC and VEGFR3 in tumor cells inducing lymphangiogenesis and metastasis, and VEGFR3 inhibition enhanced lung adenocarcinoma cell chemosensitivity through upregulation of proteins p53 and PTEN (173).

Accordingly, the Food and Drug Administration (FDA) approved several drugs targeting VEGFC/VEGFR3, such as sorafenib, sunitinib, pazopanib, and axitinib, which have given good results in the treatment of renal cell carcinoma, hepatocellular carcinoma, thyroid cancer, gastrointestinal stromal tumors and soft tissue sarcoma (174–184).

### 2.5 Endothelial-to-mesenchymal transition

Endothelial-to-mesenchymal transition (EndMT) is the phenotypic and functional transformation of endothelial to mesenchymal cells (fibroblast-like cells). During this process, ECs progressively lose their characteristic markers such as CD31, VE-cadherin, VEGFR2, and gain mesenchymal markers including fibroblast-specific protein-1 (FSP1), alpha 2 smooth muscle actin (α-SMA), vimentin and N-cadherin (185). Changes include also loss of the ability to form functional vessels, loss of cell-cell junctions and polarity, and the acquisition of cellular motility, and invasive and contractile properties (186). It occurs during embryonic development and in many pathological conditions including inflammation and cancer. Furthermore, cells in transition show a pro-inflammatory secretory phenotype related to the synthesis of extracellular matrix proteins such as fibronectin and collagens (186).

Numerous studies have demonstrated that TGF-β is the main inducer of EndMT (187–191). Other modulators of EndMT are hypoxia, EC metabolic alterations such as endothelial fatty acid oxidation and epigenetic regulators including microRNAs and long non-coding RNAs (13, 192).

EndMT has now been discovered in several pathologies, and especially in many tumors including colorectal carcinoma (193), pancreatic ductal adenocarcinoma (194), lung cancer (195), or glioblastoma (GBM) (196, 197), esophageal cancer (198), oral squamous carcinoma (199) and breast cancer (200).

During tumor development, ECs predominantly differentiate via EndMT into cancer-associated fibroblasts (CAFs) which contribute to the tumor microenvironmental plasticity and profoundly affect tumor growth and metastases (201). Moreover, EndMT participates in angiogenesis sprouting and vascular remodeling to support tumor cell dissemination, and it is involved in resistance to cancer therapy, such as chemotherapy, anti-angiogenic therapy, and radiation therapy (202). CAFs are known to produce soluble factors (IL-6 and IL-8) associated with chemoresistance and to regulate chemotherapy uptake by capturing active drugs and decreasing the expression of drug transporters. In addition, CAFs protect tumor cells from the oxidative stress induced by chemotherapy (203). Tumor vasculature EndMT-related phenotypic alterations due to fibrotic changes and specific gene loss may result in tumor resistance to and relapse after anti-VEGF therapy. Vascular damage after radiation therapy induces EndMT that reactivates dormant cancer stem cells and supports the shift of tumor associated macrophages toward an M2 phenotype, conferring tumor radioresistance (195).

## 3 Endothelial cells and tumor progression and metastasis

Among the different cell types involved in tumor progression and metastasis, tumor-associated ECs stand out for their remarkable contribution at different steps of the metastatic process including cell-cell and cell-extracellular matrix adhesions, cell invasion and/or migration and angiogenesis. Tumor-associated ECs participate in blood vessel formation which supports tumor cell growth and dissemination by providing the exit routes for metastasis in distant organs. The abnormal and leaky architecture of tumor blood vessels along with the weakened EC junctions facilitate the detachment of tumor cells from the surrounding matrix, the migration into tumor vessels via binding to the chemokine receptor CXCR7, and the metastatic spread. This metastatic process is also supported by tumor microenvironment deriving pro-inflammatory cytokines (IL-3, IL-6, and IL-8) and growth factors (bFGF, G/GM-CSF, IGF1, PDGF, and TGF-β), as well as other factors like Notch, calcineurin, biglycan, Jag1, and Slit2 (204).

TGF-β induces upregulation of the T cell inhibitory receptor T cell immunoglobulin and mucin domain-3 (TIM-3) on the EC surface and favors tumor cell proliferation, survival and migration by activating NF-κB in melanoma cells in a galectin-9-independent manner (205, 206). TIM-3 expression on lymphoma-derived ECs sustains tumor onset, growth, and dissemination by inhibiting activation of CD4^+^ T cells and Th1 polarization, and correlates with poor patient outcome (74). Moreover, TIM-3 promotes tube formation and decreases tight junction formation in vascular ECs indicating that TIM-3 expression favors tumor invasion and metastasis by inducing angiogenesis and increasing capillary permeability (207, 208).

Sustained activation of Notch1 signaling in tumor-associated ECs induces VCAM1 expression, neutrophil recruitment and a senescent, pro-inflammatory endothelium which promotes tumor cell adhesion, intravasation and metastasis. In a mouse model of ovarian carcinoma, treatment with VCAM1 and Notch 1 receptor blocking antibodies blocked Notch-driven metastasis (209). Downregulation of the tumor suppressive angiocrine factor Slit2 and upregulation of its inhibitor receptor EphA2 on tumor-associated ECs promote tumor proliferation and motility (210).

PGI2 in the tumor microenvironment can bind to and activate the peroxisome proliferator-activated receptor β/δ (PPAR β/δ) on ECs inducing EC proliferation and angiogenesis (211, 212). PPAR β/δ acts as a critical “hub node” transcription factor that shifts the angiogenic balance towards the pro-angiogenic phenotype favoring tumor development, progression, and metastasis (213–216).

Circulating tumor-associated ECs can also attach to metastasizing tumor cells protecting them from anoikis-mediated apoptosis (apoptosis in cells upon loss of attachment to the extracellular matrix) (217).

Tumor-associated ECs display high expression of B7-H3 (CD276), a critical regulator of the adaptive immune response that can distinguish between physiological and pathological angiogenesis (218, 219). B7-H3 upregulates VEGFA expression and angiogenesis by activating the NF-κB pathway in colorectal cancer and it may be a promising target for colorectal cancer treatment (220). Moreover, tumor-associated endothelial expression of B7-H3 predicts survival in ovarian carcinomas (221) and renal cell carcinomas (222). B7-H3 expression in Merkel cell carcinoma-associated ECs correlates with locally aggressive primary tumor features and increasing vascular density (223).

Tumor-associated ECs can also express CD137 (4-1BB), a member of the TNF receptor superfamily acting as a costimulatory immune receptor. Treatment of tumor-bearing immunocompromised Rag(-/-) mice with agonist CD137 monoclonal antibody not only enhances T cell activation but also stimulates tumor-associated ECs augmenting cell surface expression of ICAM-1, VCAM-1, and E-selectin with consequent promotion of CD8^+^ T cell recruitment into the malignant tissue (224).

In addition, tumor creates a local milieu favorable for tumor growth and metastasis. In high metastatic tumors, the tumor microenvironment influences EC epigenome promising upregulation of biglycan expression by DNA demethylation and enabling tumor intravasation and metastasis (225).

The tumor-associated EC altered glycosylation of surface adhesion molecules including ICAM-1, VCAM-1, and PECAM, and glycan-binding proteins (lectins) also promote tumor progression and metastasis by modifying the adhesive properties of ECs (62).

Many *in vitro* and *in vivo* studies using animal models have demonstrated the pivotal roles of galectins, a family of glycan-binding proteins, in tumor invasiveness, metastasis and angiogenesis (Reviewed in (226):. The blockade of galactin (Gal)-3 with an anti-gal-3 antibody has been shown to inhibit liver metastases of human adenocarcinoma xenotransplants in SCID mice (227). In a metastatic model, breast tumor cells expressing high levels of Gal-3 were more resistant to apoptosis induced by reactive nitrogen and oxygen species, suggesting that Gal-3 can also sustain the survival of metastasizing tumor cells (228).

Gal-1, Gal-3, and Gal-8 have been demonstrated to engage integrins or other cell surface proteins to mediate adhesion of tumor cells to extracellular matrix proteins and homotypic cell adhesion, or to inhibit adhesion favoring tumor cell detachment, dissemination through blood or lymph vessels and the attachment to ECs or basement membrane proteins at distal sites. Gal-3 produced by ECs can also foster angiogenesis by promoting EC migration and vessel formation (229–234), and by enhancing the VEGF- and basic fibroblast growth factor (bFGF)-mediated angiogenic response (235). Moreover, Gal-3 and Gal-1 directly interact with VEGFR2 increasing its pro-angiogenic function (236) and HIF1a favoring tumor progression and metastasis (237, 238).

Accordingly, it has been observed that altered galectin expression in human tumors correlates with the aggressiveness of the tumor, greater extent of vascularization and poor disease outcome (226, 239). Vaccination against Gal-1 promotes cytotoxic T cell infiltration in melanoma and reduces tumor burden in the immunized mice compared to the control group (240).

Gal-8 is markedly upregulated in inflamed human and mouse corneas and promotes VEGF-C-mediated lymphatic EC migration and sprouting. Treatment with Gal-8 inhibitors strongly reduces inflammatory lymphangiogenesis *in vivo*, suggesting Gal-8 as a promising therapeutic target for pathological lymphangiogenesis (241).

Finally, besides the impact of tumor-associated ECs on tumor growth and the metastatic process, they may affect tumor therapy resistance. The heterogeneity of tumor-associated ECs plays a central role in resistance to anti-angiogenic drugs. Treatment with anti-VEGFA antibodies and tyrosine-kinase inhibitors of VEGF receptors stimulates the production of different growth factors as alternative pro-angiogenic molecules, including angiopoietins (ANGs), epidermal growth factor (EGFs), FGFs, hepatocyte growth factor (HGF), TGFs, stromal cell-derived factor 1 (SDF1) and simultaneously, the upregulation of the respective receptors on ECs (242). EC-expressed Jag1 contributes to the development of a malignant vascular niche that is associated with an aggressive course and chemotherapy resistance (243). In addition, tumor-associated ECs produce non-conventional growth factors such as biglycan, lysyl oxidase (LOX) and pentraxin, sustaining angiogenesis processes (204). Tumor-associated ECs display cytogenetic and epigenetic abnormalities, expression of stemness genes, metabolic adaptation and sequestration of drugs in autophagic lysosomes leading to drug resistance (30, 204). Anti-VEGFA treatment and hypoxia induce glycosylation mediated resistance increasing Gal-1 production. Gal-1 binds to VEGFR2 leading to unresponsiveness to anti-VEGF therapy, and disruption of the Gal-1-VEGFR2 axis promotes vessel normalization, immune cell recruitment, tumor growth inhibition and restores sensitivity to anti-angiogenic therapy (229).

## 4 Tumor-associated endothelial cells and response to antitumor therapies

The efficacy of cancer therapy depends mainly on the ability of T cells to infiltrate tumors. The endothelium works as a barrier between the blood and the tumor. As such, it directly interacts with immune cells and plays a crucial role in recruiting and activating T cells (18).

Targeting ECs is thereby a potential strategy to optimize T cell-mediated antitumor immune responses. One strategy involves using anti-angiogenic agents to inhibit angiogenesis, overcome EC anergy, and enhance tumor T cell infiltration (244). Moreover, it has been observed that anti-angiogenic therapeutics can also normalize the dysfunctional tumor vasculature resulting in increased tumor blood perfusion and reduced tumor hypoxia, which in turn enhances drug accessibility and decreases hypoxia-mediated treatment resistance (245, 246). A single infusion of the VEGF specific antibody bevacizumab is sufficient to promote vessel normalization in rectal carcinoma patients (247). Similarly, in glioblastoma patients, treatment with cediranib, a pan-VEGF receptor tyrosine kinase inhibitor, normalizes the structure and function of the tumor vasculature increasing tumor perfusion and improving patient survival (248–251).

Preclinical studies have demonstrated that vascular normalizing doses of anti-angiogenic treatments reprogram tumor microenvironment from immunosuppressive to immunosupportive and enhance immunotherapy efficacy. For instance, low doses of an anti-VEGFR2 antibody could normalize breast tumor vasculature, redirect tumor-associated macrophages to an immune stimulatory M1-like phenotype and increase T cell tumor infiltration (252).

Immunologic approaches targeting tumor vasculature such as a model based on T cells engineered to express a chimeric antigen receptor (CAR) targeted VEGFR2 also increase tumor T cell infiltration (253). Likewise, ICI efficacy may be achieved through Th1-mediated vessel normalization indicating a mutual regulatory loop in which ICI activate IFN-γ^+^ Th1 cells that induce vessel normalization which, in turn, promotes T cell recruitment (254).

Based on these findings, combined therapeutic strategies could synergistically potentiate antitumor treatments. Combination of anti-angiogenic drugs with ICI may revert both immune and EC anergy promoting the access of cytotoxic T cells to tumors and enhancing their antitumor effects.

A dose-dependent synergism exists between anti-angiogenic therapy and ICI blockade. Low-dose anti-VEGFR2 antibody treatments can sensitize breast cancer to PD-1 blockade via upregulation of PD-1 on immune cells by stimulating the secretion of osteopontin and TGF-β (255). Treatment with a combination of anti-VEGFR2 and anti-PD-L1 antibodies induces HEV formation in pancreatic and breast tumor mouse models promoting simultaneously T cell infiltration and activation (161).

The concurrent neutralization of VEGFA and ANGPT2 by a bispecific antibody promotes vascular regression, tumor necrosis, blood vessel normalization, and increases cytotoxic T cell infiltration in both genetically engineered and transplant tumor models, including metastatic breast cancer, pancreatic neuroendocrine tumor, and melanoma. Moreover, the anti-angiogenic therapy enhances the sensitivity of mouse tumors to PD-1 blockade via upregulation of the PD-L1 expression in tumor-associated EC (256). In patients with metastatic melanoma, the combination of bevacizumab with the anti-CTLA-4 monoclonal antibody ipilimumab reverses tumor-associated EC anergy increasing the expression of the adhesion molecules E-selectin, ICAM1, and VCAM1 which enhances the recruitment of T cells in the tumor bed and improves the clinical outcome (257). The anti-PD-L1 monoclonal antibody atezolizumab in combination with bevacizumab is found to have antitumor activity with good tolerability in patients with metastatic renal cell carcinoma (258, 259), hepatocellular carcinoma (260), metastatic NSCLC without targetable mutations in association with chemotherapy (261) and metastatic NSCLC which fails to respond to atezolizumab monotherapy (262).

A novel bispecific antibody, HB0025, which concurrently blocks both the PD-L1 and VEGF pathways, has demonstrated anti-cancer activities both *in vitro* and *in vivo* and is currently under clinical trial (NCT04678908) (263, 264). An active clinical trial (NCT05116007) is ongoing to evaluate the efficacy and safety of the anti-PD-1 and -VEGF bispecific antibody AK112 combined with chemotherapy in patients with extensive stage small cell lung cancer. Furthermore, there is a multitude of ongoing clinical trials that are investigating the synergistic effect achieved through the combination of anti-angiogenic treatments with immunotherapy (**Table 2**). Encouraging preliminary results are described in various cancer types with a meager response to therapy. For instance, the combination of the anti-PD-1 monoclonal antibody pembrolizumab with the tyrosine kinase inhibitor cabozantinib improves progression free survival and overall survival compared with single-agent pembrolizumab in patients with recurrent metastatic head and neck squamous cell carcinoma. The overall response rate correlates positively with baseline CD8^+^ T cell infiltration (265).

**Table 2:** Combination of anti-angiogenic treatments with immunotherapy in ongoing clinical trials.

NCT number	Study status	Study phase	Study type	Cancer type	Drugs
NCT05285579	Recruiting	NA	Observational	Advanced/metastatic renal cell carcinoma	Tyrosine kinase inhibitors (TKI) and immune checkpoint inhibitors (ICI)
NCT04981509	Recruiting	II	Interventional	Advanced renal cell carcinoma	Bevacizumab (anti VEGF) + Atezolizumab (anti PD-L1) + Erlotinib (EGFR inhibitor)
NCT05225844	Recruiting	II	Interventional	Advanced gastrointestinal cancer	Camrelizumab (anti-PD-1) + Apatinib (VEGFR2 inhibitor)
NCT04069273	Recruiting	II	Interventional	Advanced gastric and gastroesophageal junction adenocarcinoma	Pembrolizumab (anti PD-1) + Ramucirumab (anti VEGFR2)+Paclitaxel (chemotherapeutic agent)
NCT03878472	Recruiting	II	Interventional	Resectable locally advanced gastric cancer	Camrelizumab + Apatinib
NCT05982834	Recruiting	I/II	Interventional	HER2-positive metastatic gastric cancer	Disitamab Vedotin (anti-HER2) + Fruquintinib (anti VEGFR) + Tislelizumab (anti PD-1)
NCT05372198	Recruiting	II	Interventional	Advanced colorectal cancer	Surufatinib (TKI) + immunotherapy
NCT02997228	Recruiting	III	Interventional	Metastatic colorectal cancer	Bevacizumab + Atezolizumab + mFOLFOX6 (5-Fluorouracil, modified folic acid, and oxaliplatin)
NCT05733611	Active	II	Interventional	Refractory metastatic colorectal cancer	RP2 + RP3 + bevacizumab + atezolizumab
NCT04724226	Recruiting	II	Interventional	Advanced hepatocellular carcinoma	Camrelizumab + Apatinib
NCT05313282	Recruiting	III	Interventional	C-staged hepatocellular carcinoma in BCLC classification	Hepatic Arterial Infusion + Apatinib + Camrelizumab
NCT05717400	Recruiting	IV	Interventional	Advanced hepatocellular carcinoma	Atezolizumab + Bevacizumab + Sofosbuvir (inhibitor of hepatitis C virus NS5B polymerase)
NCT04721132	Recruiting	II	Interventional	Advanced hepatocellular carcinoma	Atezolizumab + Bevacizumab
NCT03937830	Recruiting	II	Interventional	Advanced hepatocellular carcinoma	Durvalumab (anti PD-L1) + Bevacizumab, Tremelimumab (anti CTLA-4) + Transarterial Chemoembolization (TACE)
NCT04444167	Recruiting	I/II	Interventional	Advanced hepatocellular carcinoma	AK104 (anti PD-1/CTLA-4 bispecific antibody) + Lenvatinib (TKI)
NCT04443309	Recruiting	I/II	Interventional	Advanced hepatocellular carcinoma	Camrelizumab + Lenvatinib
NCT05052099	Recruiting	I/II	Interventional	Advanced biliary tract cancer	Atezolizumab + Bevacizumab + mFOLFOX6
NCT04211168	Recruiting	II	Interventional	Advanced biliary tract cancer	Toripalimab (anti PD-1)+ Lenvatinib
NCT05460195	Recruiting	II	Interventional	Resectable non-small cell lung cancer	Sintilimab (anti PD-1) + Anlotinib (VEGFR inhibitor)
NCT04973293	Recruiting	NA	Interventional	Advanced resectable non-small cell lung cancer	Bevacizumab + sintilimab + chemotherapy (carboplatin and pemetrexed)
NCT04875585	Recruiting	II	Interventional	Resectable non-small cell lung cancer	Pembrolizumab + Lenvatinib
NCT05756972	Recruiting	II/III	Interventional	Advanced non-small cell lung cancer	PM8002 (Anti-PD-L1/VEGF bispecific antibody)/Placebo + Carboplatin + Pemetrexed
NCT04749394	Recruiting	II	Interventional	Locally advanced non-small cell lung cancer	Camrelizumab + Apatinib
NCT04878107	Recruiting	II	Interventional	Metastatic non-small cell lung cancer	SBRT/LDRT + Camrelizumab + Apatinib
NCT05116007	Active	I	Interventional	Small cell lung cancer	AK112 (Anti-PD-1/VEGF bispecific antibody) + chemotherapy (Etoposide + Carboplatin)
NCT04877821	Recruiting	II	Interventional	Early-stage triple-negative breast cancer	Sintilimab + Anlotinib + Paclitaxel
NCT05549466	Recruiting	II	Interventional	Nasopharyngeal Carcinoma	Camrelizumab + Apatinib + Chemotherapy (gemcitabine/capecitabine/docetaxel)
NCT02856425	Recruiting	I	Interventional	Advanced/metastatic solid cancer	Pembrolizumab + Nintedanib (TKI)
NCT04919629	Recruiting	II	Interventional	Ovarian, fallopian tube, or primary peritoneal cancer	Bevacizumab + Pembrolizumab + Pegcetacoplan (C3 inhibitor)
NCT05063552	Recruiting	II/III	Interventional	Recurrent/metastatic head and neck cancers	Bevacizumab + Atezolizumab + Chemotherapy (carboplatin, cisplatin, docetaxel)
NCT04356729	Recruiting	II	Interventional	Melanoma	Atezolizumab + Bevacizumab
NCT03468218	Active	II	Interventional	Recurrent/metastatic head and neck cancers	Pembrolizumab + Cabozantinib (TKI)
NCT05001880	Recruiting	II	Interventional	Peritoneal malignant mesothelioma	Atezolizumab + Bevacizumab + chemotherapy (carboplatin and pemetrexed)
NCT03937635	Recruiting	III	Interventional	Smoldering multiple myeloma	Daratumumab (anti CD38) + lenalidomide (anti angiogenic)+ dexamethasone
NCT06047379	Recruiting	I/II	Interventional	Astrocytoma IDH-mutant, glioblastoma IDH-wildtype or brain metastasis	NEO212 + Ipilimumab (anti CTLA-4) + Pembrolizumab + Nivolumab (anti PD-1) + Bevacizumab + Regorafenib (anti angiogenic) + chemotherapy (carboplatin, paclitaxel, FOLFIRI Protocol)

Data were acquired from the U.S. National Library of Medicine (http://clinicaltrials.gov, accessed on December 29, 2023).

NA, Not applicable; BCLC, Barcelona clinic liver cancer; SBRT, Stereotactic body radiotherapy; LDRT, Low-dose radiotherapy.

The phase II BREAKPOINT trial (NCT03463681) met its primary endpoint showing prospective activity and safety of cabozantinib after an adjuvant or first-line ICI-based immunotherapy in patients with metastatic renal cell carcinoma (266).

Like all treatments, the combination of anti-angiogenic drugs with ICI has limitations and faces important challenges. Firstly, it is necessary to establish the appropriate drug dosage, and optimize the schedule of tumor immunotherapy and anti-angiogenesis therapy given that the dose of each drug, the sequence (simultaneous or sequential treatment), and the time of the combination can significantly impact the efficacy of the combination therapy.

Secondly, the combination of anti-angiogenic drugs with ICI has adverse effects, some of them are dose-dependent. High doses of anti-angiogenic drugs can damage tumor blood vessels resulting in hypoxia, immunosuppression and tumor treatment resistance (246). Other side effects include high-grade hypertension, decreased platelet count, proteinuria, the nonspecific activation of the immune system and hepatic toxicity (267).

Thirdly, combination therapy may not always be the best option for all patients, and there is no method to identify patients that can benefit from it. It is necessary to consider specific patient characteristics, such as the presence of autoimmune diseases or a previous organ transplantation history to avoid the nonspecific activation of the immune system that ICI treatment can cause. Specific attention should also be paid to elderly patients, patients at risk of hemorrhages, and patients suffering from severe cardiovascular diseases, hepatopathies or gastrointestinal problems as they may be exposed to a higher risk of exacerbations (268).

Furthermore, there is a lack of validated predictive biomarkers that can allow patient selection and stratification, and guide treatment decisions before and during therapy. Recent studies have suggested PD-L1 expression as a potential biomarker; they have reported that ICI combined with angiogenesis inhibitors improved the outcome in patients with positive PD-L1 expression affected by renal cell carcinoma, NSCLC, advanced HER2-positive breast cancer and untreated, unresectable hepatocellular carcinoma (260, 269–273). Moreover, pre-existing immunity (high expression of CD274, T cell effector signature and intratumoral CD8^+^ T cell density), high expression of Treg, MDSC and VEGFR2 in tumor tissue could also be potential candidate biomarkers for prediction of response and resistance to ICI and anti-angiogenic combination therapy (274). In addition to biomarkers derived from analysis of tumor tissue, circulating biomarkers, such as levels of VEGFA, soluble VEGFR2 and circulating ECs could be considered to monitor treatment responses. Moreover, non-invasive imaging biomarkers, including computed tomography (CT)-based perfusion scan, dynamic contrast-enhanced ultrasonography, dynamic contrast-enhanced magnetic resonance imaging and magnetic resonance imaging-based diffusion-weighted imaging and perfusion could be helpful to examine tumor vessel perfusion and vascularity during treatment (275). Increasing evidence suggests a role of the composition and function of the gut microbiome as a predictive biomarker (276). Based on these findings, a new approach in cancer management should aim to discover a composite biomarker of response to therapy that uses data from imaging, tumor tissue-derived, circulating and gut microbiome-derived biomarkers. The identification of a composite biomarker, that can be easily and continuously monitored, may support the assessment of treatment efficacy and the development of a personalized treatment based on patient-customized adjustment of therapeutic regimens.

Alternative strategies target tumor-derived extracellular vesicles (EVs) carrying pro-angiogenic factors such as VEGF, IL-6, microRNAs which stimulate EC functions or mediate drug resistance (277, 278). Recent studies have tried to improve the efficacy of anti‐angiogenic therapies by blocking tumor-derived EVs. For instance, patients with elevated EV‐VEGF levels can be treated with VEGF‐binding protein inhibitors or VEGFR2‐neutralizing antibodies to block EV-VEGF which are resistant to bevacizumab treatment (278).

Finally, a study has investigated the use of nanotechnology-based approaches to target ECs. The nanoformulated STING activator ZnCDA activates ECs resulting in disruption of tumor vasculature, increase of tumor-targeted drug accumulation and improvement of tumor-associated macrophage functions (279).

## 5 Conclusions

ECs play a critical role in tumor development which has gone beyond angiogenesis. During tumor progression, multiple cellular pathways can interact and regulate each other through signal competition, redundancy, shared downstream signaling network, and many crosstalk and cross-regulation mechanisms. Understanding the effects of ECs on tumor progression and their interactions with the other components of the tumor microenvironment requires computational models that integrate multiple cell types.

ECs are now considered active participants in the tumor microenvironment, secreting angiogenic factors such as cytokines, growth factors, and chemokines, and interacting with immune cells. Crosstalk between tumor endothelium and immune cells strongly impacts the immune response to tumors and contributes to immunosuppression by downregulation of antigen presentation and recruitment of immune effector cells. Moreover, tumor-associated ECs support the expansion of immunosuppressive cell populations, such as Treg cells, contributing to tumor immune escape. In addition, tumor-associated ECs of blood and lymphatic vessels are directly involved in the formation of distant metastasis. Emerging therapeutic strategies aim at modulating both ECs and immune cells, not only to block angiogenesis but also to enhance the recruitment and activation of effector cells within the tumor. A combination of anti-angiogenic treatments with immunotherapy strategies, including ICI, CAR T cells, and bispecific antibodies has emerged. Many pivotal clinical trials have proven the validity of these combinations which are now successfully used in patients with metastatic melanoma, renal cell carcinoma, hepatocellular carcinoma and metastatic NSCLC. Moreover, a multitude of clinical trials focusing on these combined therapeutic strategies are currently ongoing and new studies aimed at evaluating the use of nanotechnology-based approaches to target ECs have emerged.

Understanding the interplay between ECs, immune cells and tumor cells can provide important insights into the mechanisms of tumor progression and help overcome the limitations of current therapeutic interventions, including the emergence of treatment resistance and the mechanisms of immune escape.

## Author contributions

PL: Conceptualization, Data curation, Writing – original draft. EM: Data curation, Writing – review & editing. NS: Data curation, Writing – review & editing. EF: Data curation, Writing – review & editing. FP: Data curation, Writing – review & editing. GB: Data curation, Writing – review & editing. MP: Data curation, Writing – review & editing. VR: Conceptualization, Supervision, Writing – review & editing.

## References

[1] Hennigs JK Matuszcak C Trepel M Korbelin J. Vascular endothelial cells: heterogeneity and targeting approaches. Cells (2021) 10:2712–51. doi: 10.3390/cells10102712 PMC8534745 34685692

[2] Aird WC. Endothelial cell heterogeneity. Cold Spring Harb Perspect Med (2012) 2:a006429. doi: 10.1101/cshperspect.a006429 22315715 PMC3253027

[3] Kruger-Genge A Blocki A Franke RP Jung F. Vascular endothelial cell biology: an update. Int J Mol Sci (2019) 20:4411–33. doi: 10.3390/ijms20184411 PMC6769656 31500313

[4] Pusztaszeri MP Seelentag W Bosman FT. Immunohistochemical expression of endothelial markers cd31, cd34, von willebrand factor, and fli-1 in normal human tissues. J Histochem Cytochem (2006) 54:385–95. doi: 10.1369/jhc.4A6514.2005 16234507

[5] Reinhart WH. Shear-dependence of endothelial functions. Experientia (1994) 50:87–93. doi: 10.1007/BF01984940 8125177

[6] Resnick N Gimbrone MA Jr. Hemodynamic forces are complex regulators of endothelial gene expression. FASEB J (1995) 9:874–82. doi: 10.1096/fasebj.9.10.7615157 7615157

[7] Shao Y Saredy J Yang WY Sun Y Lu Y Saaoud F. Vascular endothelial cells and innate immunity. Arterioscler Thromb Vasc Biol (2020) 40:e138–e52. doi: 10.1161/ATVBAHA.120.314330 PMC7263359 32459541

[8] Schaaf MB Houbaert D Mece O Agostinis P. Autophagy in endothelial cells and tumor angiogenesis. Cell Death Differ (2019) 26:665–79. doi: 10.1038/s41418-019-0287-8 PMC6460396 30692642

[9] Morikawa S Baluk P Kaidoh T Haskell A Jain RK McDonald DM. Abnormalities in pericytes on blood vessels and endothelial sprouts in tumors. Am J Pathol (2002) 160:985–1000. doi: 10.1016/S0002-9440(10)64920-6 11891196 PMC1867175

[10] Nagl L Horvath L Pircher A Wolf D. Tumor endothelial cells (Tecs) as potential immune directors of the tumor microenvironment - new findings and future perspectives. Front Cell Dev Biol (2020) 8:766. doi: 10.3389/fcell.2020.00766 32974337 PMC7466447

[11] St Croix B Rago C Velculescu V Traverso G Romans KE Montgomery E. Genes expressed in human tumor endothelium. Science (2000) 289:1197–202. doi: 10.1126/science.289.5482.1197 10947988

[12] Dudley AC. Tumor endothelial cells. Cold Spring Harb Perspect Med (2012) 2:a006536. doi: 10.1101/cshperspect.a006536 22393533 PMC3282494

[13] Clere N Renault S Corre I. Endothelial-to-mesenchymal transition in cancer. Front Cell Dev Biol (2020) 8:747. doi: 10.3389/fcell.2020.00747 32923440 PMC7456955

[14] Goveia J Rohlenova K Taverna F Treps L Conradi LC Pircher A. An integrated gene expression landscape profiling approach to identify lung tumor endothelial cell heterogeneity and angiogenic candidates. Cancer Cell (2020) 37:21–36 e13. doi: 10.1016/j.ccell.2019.12.001 31935371

[15] Lambrechts D Wauters E Boeckx B Aibar S Nittner D Burton O. Phenotype molding of stromal cells in the lung tumor microenvironment. Nat Med (2018) 24:1277–89. doi: 10.1038/s41591-018-0096-5 29988129

[16] Rohlenova K Goveia J Garcia-Caballero M Subramanian A Kalucka J Treps L. Single-cell rna sequencing maps endothelial metabolic plasticity in pathological angiogenesis. Cell Metab (2020) 31:862–77 e14. doi: 10.1016/j.cmet.2020.03.009 32268117

[17] Jing X Yang F Shao C Wei K Xie M Shen H. Role of hypoxia in cancer therapy by regulating the tumor microenvironment. Mol Cancer (2019) 18:157. doi: 10.1186/s12943-019-1089-9 31711497 PMC6844052

[18] Fang J Lu Y Zheng J Jiang X Shen H Shang X. Exploring the crosstalk between endothelial cells, immune cells, and immune checkpoints in the tumor microenvironment: new insights and therapeutic implications. Cell Death Dis (2023) 14:586. doi: 10.1038/s41419-023-06119-x 37666809 PMC10477350

[19] Dieu-Nosjean MC Giraldo NA Kaplon H Germain C Fridman WH Sautes-Fridman C. Tertiary lymphoid structures, drivers of the anti-tumor responses in human cancers. Immunol Rev (2016) 271:260–75. doi: 10.1111/imr.12405 27088920

[20] Dayan F Mazure NM Brahimi-Horn MC Pouyssegur J. A dialogue between the hypoxia-inducible factor and the tumor microenvironment. Cancer Microenviron (2008) 1:53–68. doi: 10.1007/s12307-008-0006-3 19308685 PMC2654353

[21] Leone P Buonavoglia A Fasano R Solimando AG De Re V Cicco S. Insights into the regulation of tumor angiogenesis by micro-rnas. J Clin Med (2019) 8:2030–50. doi: 10.3390/jcm8122030 PMC6947031 31757094

[22] Zhang Y Wang H Oliveira RHM Zhao C Popel AS. Systems biology of angiogenesis signaling: computational models and omics. WIREs Mech Dis (2022) 14:e1550. doi: 10.1002/wsbm.1550 34970866 PMC9243197

[23] Carmeliet P Jain RK. Angiogenesis in cancer and other diseases. Nature (2000) 407:249–57. doi: 10.1038/35025220 11001068

[24] Potente M Gerhardt H Carmeliet P. Basic and therapeutic aspects of angiogenesis. Cell (2011) 146:873–87. doi: 10.1016/j.cell.2011.08.039 21925313

[25] Bergers G Benjamin LE. Tumorigenesis and the angiogenic switch. Nat Rev Cancer (2003) 3:401–10. doi: 10.1038/nrc1093 12778130

[26] Matsuda K Ohga N Hida Y Muraki C Tsuchiya K Kurosu T. Isolated tumor endothelial cells maintain specific character during long-term culture. Biochem Biophys Res Commun (2010) 394:947–54. doi: 10.1016/j.bbrc.2010.03.089 20302845

[27] Ohga N Ishikawa S Maishi N Akiyama K Hida Y Kawamoto T. Heterogeneity of tumor endothelial cells: comparison between tumor endothelial cells isolated from high- and low-metastatic tumors. Am J Pathol (2012) 180:1294–307. doi: 10.1016/j.ajpath.2011.11.035 22245217

[28] Hojo T Maishi N Towfik AM Akiyama K Ohga N Shindoh M. Ros enhance angiogenic properties via regulation of nrf2 in tumor endothelial cells. Oncotarget (2017) 8:45484–95. doi: 10.18632/oncotarget.17567 PMC5542202 28525375

[29] Kondoh M Ohga N Akiyama K Hida Y Maishi N Towfik AM. Hypoxia-induced reactive oxygen species cause chromosomal abnormalities in endothelial cells in the tumor microenvironment. PloS One (2013) 8:e80349. doi: 10.1371/journal.pone.0080349 24260373 PMC3829944

[30] Hida K Hida Y Amin DN Flint AF Panigrahy D Morton CC. Tumor-associated endothelial cells with cytogenetic abnormalities. Cancer Res (2004) 64:8249–55. doi: 10.1158/0008-5472.CAN-04-1567 15548691

[31] Ciesielski O Biesiekierska M Panthu B Vialichka V Pirola L Balcerczyk A. The epigenetic profile of tumor endothelial cells. Effects of combined therapy with antiangiogenic and epigenetic drugs on cancer progression. Int J Mol Sci (2020) 21:2606–28. doi: 10.3390/ijms21072606 PMC7177242 32283668

[32] Cantelmo AR Conradi LC Brajic A Goveia J Kalucka J Pircher A. Inhibition of the glycolytic activator pfkfb3 in endothelium induces tumor vessel normalization, impairs metastasis, and improves chemotherapy. Cancer Cell (2016) 30:968–85. doi: 10.1016/j.ccell.2016.10.006 PMC5675554 27866851

[33] Lin CY Loven J Rahl PB Paranal RM Burge CB Bradner JE. Transcriptional amplification in tumor cells with elevated C-myc. Cell (2012) 151:56–67. doi: 10.1016/j.cell.2012.08.026 23021215 PMC3462372

[34] Baudino TA McKay C Pendeville-Samain H Nilsson JA Maclean KH White EL. C-myc is essential for vasculogenesis and angiogenesis during development and tumor progression. Genes Dev (2002) 16:2530–43. doi: 10.1101/gad.1024602 PMC187450 12368264

[35] Sievert W Tapio S Breuninger S Gaipl U Andratschke N Trott KR. Adhesion molecule expression and function of primary endothelial cells in benign and Malignant tissues correlates with proliferation. PloS One (2014) 9:e91808. doi: 10.1371/journal.pone.0091808 24632811 PMC3954738

[36] Naschberger E Schellerer VS Rau TT Croner RS Sturzl M. Isolation of endothelial cells from human tumors. Methods Mol Biol (2011) 731:209–18. doi: 10.1007/978-1-61779-080-5_18 21516410

[37] Blanco R Gerhardt H. Vegf and notch in tip and stalk cell selection. Cold Spring Harb Perspect Med (2013) 3:a006569. doi: 10.1101/cshperspect.a006569 23085847 PMC3530037

[38] Naito H Kidoya H Sakimoto S Wakabayashi T Takakura N. Identification and characterization of a resident vascular stem/progenitor cell population in preexisting blood vessels. EMBO J (2012) 31:842–55. doi: 10.1038/emboj.2011.465 PMC3280559 22179698

[39] Wakabayashi T Naito H Suehiro JI Lin Y Kawaji H Iba T. Cd157 marks tissue-resident endothelial stem cells with homeostatic and regenerative properties. Cell Stem Cell (2018) 22:384–97 e6. doi: 10.1016/j.stem.2018.01.010 29429943

[40] Yao X Zeng Y. Tumour associated endothelial cells: origin, characteristics and role in metastasis and anti-angiogenic resistance. Front Physiol (2023) 14:1199225. doi: 10.3389/fphys.2023.1199225 37389120 PMC10301839

[41] Hirschi KK Ingram DA Yoder MC. Assessing identity, phenotype, and fate of endothelial progenitor cells. Arterioscler Thromb Vasc Biol (2008) 28:1584–95. doi: 10.1161/ATVBAHA.107.155960 PMC5244813 18669889

[42] Wang R Chadalavada K Wilshire J Kowalik U Hovinga KE Geber A. Glioblastoma stem-like cells give rise to tumour endothelium. Nature (2010) 468:829–33. doi: 10.1038/nature09624 21102433

[43] Patel J Seppanen EJ Rodero MP Wong HY Donovan P Neufeld Z. Functional definition of progenitors versus mature endothelial cells reveals key soxf-dependent differentiation process. Circulation (2017) 135:786–805. doi: 10.1161/CIRCULATIONAHA.116.024754 27899395

[44] Hida K Maishi N Akiyama K Ohmura-Kakutani H Torii C Ohga N. Tumor endothelial cells with high aldehyde dehydrogenase activity show drug resistance. Cancer Sci (2017) 108:2195–203. doi: 10.1111/cas.13388 PMC5666026 28851003

[45] Ohmura-Kakutani H Akiyama K Maishi N Ohga N Hida Y Kawamoto T. Identification of tumor endothelial cells with high aldehyde dehydrogenase activity and a highly angiogenic phenotype. PloS One (2014) 9:e113910. doi: 10.1371/journal.pone.0113910 25437864 PMC4250080

[46] Ge H Luo H. Overview of advances in vasculogenic mimicry - a potential target for tumor therapy. Cancer Manag Res (2018) 10:2429–37. doi: 10.2147/CMAR.S164675 PMC6080880 30122992

[47] Streubel B Chott A Huber D Exner M Jager U Wagner O. Lymphoma-specific genetic aberrations in microvascular endothelial cells in B-cell lymphomas. N Engl J Med (2004) 351:250–9. doi: 10.1056/NEJMoa033153 15254283

[48] Chen H Campbell RA Chang Y Li M Wang CS Li J. Pleiotrophin produced by multiple myeloma induces transdifferentiation of monocytes into vascular endothelial cells: A novel mechanism of tumor-induced vasculogenesis. Blood (2009) 113:1992–2002. doi: 10.1182/blood-2008-02-133751 19060246 PMC2651013

[49] Fernandez Pujol B Lucibello FC Zuzarte M Lutjens P Muller R Havemann K. Dendritic cells derived from peripheral monocytes express endothelial markers and in the presence of angiogenic growth factors differentiate into endothelial-like cells. Eur J Cell Biol (2001) 80:99–110. doi: 10.1078/0171-9335-00136 11211940

[50] Amersfoort J Eelen G Carmeliet P. Immunomodulation by endothelial cells - partnering up with the immune system? Nat Rev Immunol (2022) 22:576–88. doi: 10.1038/s41577-022-00694-4 PMC8920067 35288707

[51] Carman CV Martinelli R. T lymphocyte-endothelial interactions: emerging understanding of trafficking and antigen-specific immunity. Front Immunol (2015) 6:603. doi: 10.3389/fimmu.2015.00603 26635815 PMC4657048

[52] De Sanctis F Ugel S Facciponte J Facciabene A. The dark side of tumor-associated endothelial cells. Semin Immunol (2018) 35:35–47. doi: 10.1016/j.smim.2018.02.002 29490888

[53] Ley K Laudanna C Cybulsky MI Nourshargh S. Getting to the site of inflammation: the leukocyte adhesion cascade updated. Nat Rev Immunol (2007) 7:678–89. doi: 10.1038/nri2156 17717539

[54] Harjunpaa H Llort Asens M Guenther C Fagerholm SC. Cell adhesion molecules and their roles and regulation in the immune and tumor microenvironment. Front Immunol (2019) 10:1078. doi: 10.3389/fimmu.2019.01078 31231358 PMC6558418

[55] De Caterina R Libby P Peng HB Thannickal VJ Rajavashisth TB Gimbrone MA Jr.. Nitric oxide decreases cytokine-induced endothelial activation. Nitric oxide selectively reduces endothelial expression of adhesion molecules and proinflammatory cytokines. J Clin Invest (1995) 96:60–8. doi: 10.1172/JCI118074 PMC185173 7542286

[56] He Q Jamalpour M Bergquist E Anderson RL Gustafsson K Welsh M. Mouse breast carcinoma monocytic/macrophagic myeloid-derived suppressor cell infiltration as a consequence of endothelial dysfunction in shb-deficient endothelial cells increases tumor lung metastasis. Int J Mol Sci (2021) 22:11478–92. doi: 10.3390/ijms222111478 PMC8583852 34768912

[57] Facciabene A Peng X Hagemann IS Balint K Barchetti A Wang LP. Tumour hypoxia promotes tolerance and angiogenesis via ccl28 and T(Reg) cells. Nature (2011) 475:226–30. doi: 10.1038/nature10169 21753853

[58] Irjala H Elima K Johansson EL Merinen M Kontula K Alanen K. The same endothelial receptor controls lymphocyte traffic both in vascular and lymphatic vessels. Eur J Immunol (2003) 33:815–24. doi: 10.1002/eji.200323859 12616502

[59] Karikoski M Marttila-Ichihara F Elima K Rantakari P Hollmen M Kelkka T. Clever-1/stabilin-1 controls cancer growth and metastasis. Clin Cancer Res (2014) 20:6452–64. doi: 10.1158/1078-0432.CCR-14-1236 25320356

[60] Martens JH Kzhyshkowska J Falkowski-Hansen M Schledzewski K Gratchev A Mansmann U. Differential expression of a gene signature for scavenger/lectin receptors by endothelial cells and macrophages in human lymph node sinuses, the primary sites of regional metastasis. J Pathol (2006) 208:574–89. doi: 10.1002/path.1921 16440291

[61] Prevo R Banerji S Ni J Jackson DG. Rapid plasma membrane-endosomal trafficking of the lymph node sinus and high endothelial venule scavenger receptor/homing receptor stabilin-1 (Feel-1/clever-1). J Biol Chem (2004) 279:52580–92. doi: 10.1074/jbc.M406897200 15345716

[62] Chandler KB Costello CE Rahimi N. Glycosylation in the tumor microenvironment: implications for tumor angiogenesis and metastasis. Cells (2019) 8:544–65. doi: 10.3390/cells8060544 PMC6627046 31195728

[63] Griffioen AW Damen CA Blijham GH Groenewegen G. Tumor angiogenesis is accompanied by a decreased inflammatory response of tumor-associated endothelium. Blood (1996) 88:667–73. 8695814

[64] Huang H Langenkamp E Georganaki M Loskog A Fuchs PF Dieterich LC. Vegf suppresses T-lymphocyte infiltration in the tumor microenvironment through inhibition of nf-kappab-induced endothelial activation. FASEB J (2015) 29:227–38. doi: 10.1096/fj.14-250985 25361735

[65] Thompson TW Kim AB Li PJ Wang J Jackson BT Huang KTH. Endothelial cells express nkg2d ligands and desensitize antitumor nk responses. Elife (2017) 6:e30881–902. doi: 10.7554/eLife.30881 29231815 PMC5792093

[66] Won Jun H Kyung Lee H Ho Na I Jeong Lee S Kim K Park G. The role of ccl2, ccl7, icam-1, and vcam-1 in interaction of endothelial cells and natural killer cells. Int Immunopharmacol (2022) 113:109332. doi: 10.1016/j.intimp.2022.109332 36274485

[67] Doak GR Schwertfeger KL Wood DK. Distant relations: macrophage functions in the metastatic niche. Trends Cancer (2018) 4:445–59. doi: 10.1016/j.trecan.2018.03.011 PMC5990045 29860988

[68] Wang X Zhao X Wang K Wu L Duan T. Interaction of monocytes/macrophages with ovarian cancer cells promotes angiogenesis in vitro. Cancer Sci (2013) 104:516–23. doi: 10.1111/cas.12110 PMC7657218 23347208

[69] Waugh DJ Wilson C. The interleukin-8 pathway in cancer. Clin Cancer Res (2008) 14:6735–41. doi: 10.1158/1078-0432.CCR-07-4843 18980965

[70] Yeo EJ Cassetta L Qian BZ Lewkowich I Li JF Stefater JA 3rd. Myeloid wnt7b mediates the angiogenic switch and metastasis in breast cancer. Cancer Res (2014) 74:2962–73. doi: 10.1158/0008-5472.CAN-13-2421 PMC4137408 24638982

[71] Dalton HJ Armaiz-Pena GN Gonzalez-Villasana V Lopez-Berestein G Bar-Eli M Sood AK. Monocyte subpopulations in angiogenesis. Cancer Res (2014) 74:1287–93. doi: 10.1158/0008-5472.CAN-13-2825 PMC4040005 24556724

[72] Hellenthal KEM Brabenec L Wagner NM. Regulation and dysregulation of endothelial permeability during systemic inflammation. Cells (2022) 11:1935–56. doi: 10.3390/cells11121935 PMC9221661 35741064

[73] Wu Q Wu X Ying X Zhu Q Wang X Jiang L. Suppression of endothelial cell migration by tumor associated macrophage-derived exosomes is reversed by epithelial ovarian cancer exosomal lncrna. Cancer Cell Int (2017) 17:62. doi: 10.1186/s12935-017-0430-x 28592924 PMC5461704

[74] Huang X Bai X Cao Y Wu J Huang M Tang D. Lymphoma endothelium preferentially expresses tim-3 and facilitates the progression of lymphoma by mediating immune evasion. J Exp Med (2010) 207:505–20. doi: 10.1084/jem.20090397 PMC2839144 20176801

[75] Yang Y Guo Z Chen W Wang X Cao M Han X. M2 macrophage-derived exosomes promote angiogenesis and growth of pancreatic ductal adenocarcinoma by targeting E2f2. Mol Ther (2021) 29:1226–38. doi: 10.1016/j.ymthe.2020.11.024 PMC7934635 33221435

[76] Bieniasz-Krzywiec P Martin-Perez R Ehling M Garcia-Caballero M Pinioti S Pretto S. Podoplanin-expressing macrophages promote lymphangiogenesis and lymphoinvasion in breast cancer. Cell Metab (2019) 30:917–36 e10. doi: 10.1016/j.cmet.2019.07.015 31447322 PMC7616630

[77] Gao L Chen K Gao Q Wang X Sun J Yang YG. Cd47 deficiency in tumor stroma promotes tumor progression by enhancing angiogenesis. Oncotarget (2017) 8:22406–13. doi: 10.18632/oncotarget.9899 PMC5410232 27283989

[78] Maxhimer JB Soto-Pantoja DR Ridnour LA Shih HB Degraff WG Tsokos M. Radioprotection in normal tissue and delayed tumor growth by blockade of cd47 signaling. Sci Transl Med (2009) 1:3ra7. doi: 10.1126/scitranslmed.3000139 PMC2811586 20161613

[79] Cols M Barra CM He B Puga I Xu W Chiu A. Stromal endothelial cells establish a bidirectional crosstalk with chronic lymphocytic leukemia cells through the tnf-related factors baff, april, and cd40l. J Immunol (2012) 188:6071–83. doi: 10.4049/jimmunol.1102066 PMC3370079 22593611

[80] Shen Y Wang X Liu Y Singhal M Gurkaslar C Valls AF. Stat3-yap/taz signaling in endothelial cells promotes tumor angiogenesis. Sci Signal (2021) 14:eabj8393. doi: 10.1126/scisignal.abj8393 34874746

[81] Yang C Lee H Pal S Jove V Deng J Zhang W. B cells promote tumor progression via stat3 regulated-angiogenesis. PloS One (2013) 8:e64159. doi: 10.1371/journal.pone.0064159 23734190 PMC3667024

[82] Kam NW Wu KC Dai W Wang Y Yan LYC Shakya R. Peritumoral B cells drive proangiogenic responses in hmgb1-enriched esophageal squamous cell carcinoma. Angiogenesis (2022) 25:181–203. doi: 10.1007/s10456-021-09819-0 34617194 PMC8494172

[83] Zhao Q Han YM Song P Liu Z Yuan Z Zou MH. Endothelial cell-specific expression of serine/threonine kinase 11 modulates dendritic cell differentiation. Nat Commun (2022) 13:648. doi: 10.1038/s41467-022-28316-6 35115536 PMC8814147

[84] Gottfried E Kreutz M Haffner S Holler E Iacobelli M Andreesen R. Differentiation of human tumour-associated dendritic cells into endothelial-like cells: an alternative pathway of tumour angiogenesis. Scand J Immunol (2007) 65:329–35. doi: 10.1111/j.1365-3083.2007.01903.x 17386023

[85] Lu J Zhao J Liu K Zhao J Yang H Huang Y. Mapk/erk1/2 signaling mediates endothelial-like differentiation of immature dcs in the microenvironment of esophageal squamous cell carcinoma. Cell Mol Life Sci (2010) 67:2091–106. doi: 10.1007/s00018-010-0316-8 PMC11115913 20221785

[86] Ammendola M Leporini C Marech I Gadaleta CD Scognamillo G Sacco R. Targeting mast cells tryptase in tumor microenvironment: A potential antiangiogenetic strategy. BioMed Res Int (2014) 2014:154702. doi: 10.1155/2014/154702 25295247 PMC4177740

[87] Demaria O De Gassart A Coso S Gestermann N Di Domizio J Flatz L. Sting activation of tumor endothelial cells initiates spontaneous and therapeutic antitumor immunity. Proc Natl Acad Sci USA (2015) 112:15408–13. doi: 10.1073/pnas.1512832112 PMC4687570 26607445

[88] Li X Shu C Yi G Chaton CT Shelton CL Diao J. Cyclic gmp-amp synthase is activated by double-stranded DNA-induced oligomerization. Immunity (2013) 39:1019–31. doi: 10.1016/j.immuni.2013.10.019 PMC3886715 24332030

[89] Berger G Knelson EH Jimenez-Macias JL Nowicki MO Han S Panagioti E. Sting activation promotes robust immune response and nk cell-mediated tumor regression in glioblastoma models. Proc Natl Acad Sci USA (2022) 119:e2111003119. doi: 10.1073/pnas.2111003119 35787058 PMC9282249

[90] Berger G Marloye M Lawler SE. Pharmacological modulation of the sting pathway for cancer immunotherapy. Trends Mol Med (2019) 25:412–27. doi: 10.1016/j.molmed.2019.02.007 30885429

[91] Chen YP Xu L Tang TW Chen CH Zheng QH Liu TP. Sting activator C-di-gmp-loaded mesoporous silica nanoparticles enhance immunotherapy against breast cancer. ACS Appl Mater Interfaces (2020) 12:56741–52. doi: 10.1021/acsami.0c16728 33305564

[92] Ohkuri T Ghosh A Kosaka A Zhu J Ikeura M David M. Sting contributes to antiglioma immunity via triggering type I ifn signals in the tumor microenvironment. Cancer Immunol Res (2014) 2:1199–208. doi: 10.1158/2326-6066.CIR-14-0099 PMC4258479 25300859

[93] An M Yu C Xi J Reyes J Mao G Wei WZ. Induction of necrotic cell death and activation of sting in the tumor microenvironment via cationic silica nanoparticles leading to enhanced antitumor immunity. Nanoscale (2018) 10:9311–9. doi: 10.1039/c8nr01376d PMC5969905 29737353

[94] Yang H Lee WS Kong SJ Kim CG Kim JH Chang SK. Sting activation reprograms tumor vasculatures and synergizes with vegfr2 blockade. J Clin Invest (2019) 129:4350–64. doi: 10.1172/JCI125413 PMC6763266 31343989

[95] Sakano Y Noda T Kobayashi S Sasaki K Iwagami Y Yamada D. Tumor endothelial cell-induced cd8(+) T-cell exhaustion via gpnmb in hepatocellular carcinoma. Cancer Sci (2022) 113:1625–38. doi: 10.1111/cas.15331 PMC9128167 35289033

[96] Georganaki M Ramachandran M Tuit S Nunez NG Karampatzakis A Fotaki G. Tumor endothelial cell up-regulation of ido1 is an immunosuppressive feed-back mechanism that reduces the response to cd40-stimulating immunotherapy. Oncoimmunology (2020) 9:1730538. doi: 10.1080/2162402X.2020.1730538 32231867 PMC7094447

[97] Munn DH Mellor AL. Ido in the tumor microenvironment: inflammation, counter-regulation, and tolerance. Trends Immunol (2016) 37:193–207. doi: 10.1016/j.it.2016.01.002 26839260 PMC4916957

[98] Fujiwara Y Kato S Nesline MK Conroy JM DePietro P Pabla S. Indoleamine 2,3-dioxygenase (Ido) inhibitors and cancer immunotherapy. Cancer Treat Rev (2022) 110:102461. doi: 10.1016/j.ctrv.2022.102461 36058143 PMC12187009

[99] Le Naour J Galluzzi L Zitvogel L Kroemer G Vacchelli E. Trial watch: ido inhibitors in cancer therapy. Oncoimmunology (2020) 9:1777625. doi: 10.1080/2162402X.2020.1777625 32934882 PMC7466863

[100] Motz GT Santoro SP Wang LP Garrabrant T Lastra RR Hagemann IS. Tumor endothelium fasl establishes a selective immune barrier promoting tolerance in tumors. Nat Med (2014) 20:607–15. doi: 10.1038/nm.3541 PMC4060245 24793239

[101] Leone P Di Lernia G Solimando AG Cicco S Saltarella I Lamanuzzi A. Bone marrow endothelial cells sustain a tumor-specific cd8(+) T cell subset with suppressive function in myeloma patients. Oncoimmunology (2019) 8:e1486949. doi: 10.1080/2162402X.2018.1486949 30546939 PMC6287798

[102] Lim WC Olding M Healy E Millar TM. Human endothelial cells modulate cd4(+) T cell populations and enhance regulatory T cell suppressive capacity. Front Immunol (2018) 9:565. doi: 10.3389/fimmu.2018.00565 29628925 PMC5876242

[103] Zheng Z Xu PP Wang L Zhao HJ Weng XQ Zhong HJ. Mir21 sensitized B-lymphoma cells to abt-199 via icos/icosl-mediated interaction of treg cells with endothelial cells. J Exp Clin Cancer Res (2017) 36:82. doi: 10.1186/s13046-017-0551-z 28637496 PMC5480196

[104] Raineri D Dianzani C Cappellano G Maione F Baldanzi G Iacobucci I. Osteopontin binds icosl promoting tumor metastasis. Commun Biol (2020) 3:615. doi: 10.1038/s42003-020-01333-1 33106594 PMC7588454

[105] Dieu-Nosjean MC Goc J Giraldo NA Sautes-Fridman C Fridman WH. Tertiary lymphoid structures in cancer and beyond. Trends Immunol (2014) 35:571–80. doi: 10.1016/j.it.2014.09.006 25443495

[106] Link A Hardie DL Favre S Britschgi MR Adams DH Sixt M. Association of T-zone reticular networks and conduits with ectopic lymphoid tissues in mice and humans. Am J Pathol (2011) 178:1662–75. doi: 10.1016/j.ajpath.2010.12.039 PMC3070229 21435450

[107] Penaranda C Tang Q Ruddle NH Bluestone JA. Prevention of diabetes by fty720-mediated stabilization of peri-islet tertiary lymphoid organs. Diabetes (2010) 59:1461–8. doi: 10.2337/db09-1129 PMC2874707 20299465

[108] Stranford S Ruddle NH. Follicular dendritic cells, conduits, lymphatic vessels, and high endothelial venules in tertiary lymphoid organs: parallels with lymph node stroma. Front Immunol (2012) 3:350. doi: 10.3389/fimmu.2012.00350 23230435 PMC3515885

[109] Ruddle NH. Lymphatic vessels and tertiary lymphoid organs. J Clin Invest (2014) 124:953–9. doi: 10.1172/JCI71611 PMC3934190 24590281

[110] Meier D Bornmann C Chappaz S Schmutz S Otten LA Ceredig R. Ectopic lymphoid-organ development occurs through interleukin 7-mediated enhanced survival of lymphoid-tissue-inducer cells. Immunity (2007) 26:643–54. doi: 10.1016/j.immuni.2007.04.009 17521585

[111] Colbeck EJ Ager A Gallimore A Jones GW. Tertiary lymphoid structures in cancer: drivers of antitumor immunity, immunosuppression, or bystander sentinels in disease? Front Immunol (2017) 8:1830. doi: 10.3389/fimmu.2017.01830 29312327 PMC5742143

[112] Deteix C Attuil-Audenis V Duthey A Patey N McGregor B Dubois V. Intragraft th17 infiltrate promotes lymphoid neogenesis and hastens clinical chronic rejection. J Immunol (2010) 184:5344–51. doi: 10.4049/jimmunol.0902999 20357253

[113] Guedj K Khallou-Laschet J Clement M Morvan M Gaston AT Fornasa G. M1 macrophages act as ltbetar-independent lymphoid tissue inducer cells during atherosclerosis-related lymphoid neogenesis. Cardiovasc Res (2014) 101:434–43. doi: 10.1093/cvr/cvt263 24272771

[114] Lochner M Ohnmacht C Presley L Bruhns P Si-Tahar M Sawa S. Microbiota-induced tertiary lymphoid tissues aggravate inflammatory disease in the absence of rorgamma T and lti cells. J Exp Med (2011) 208:125–34. doi: 10.1084/jem.20100052 PMC3023125 21173107

[115] Peters A Pitcher LA Sullivan JM Mitsdoerffer M Acton SE Franz B. Th17 cells induce ectopic lymphoid follicles in central nervous system tissue inflammation. Immunity (2011) 35:986–96. doi: 10.1016/j.immuni.2011.10.015 PMC3422678 22177922

[116] Furtado GC Marinkovic T Martin AP Garin A Hoch B Hubner W. Lymphotoxin beta receptor signaling is required for inflammatory lymphangiogenesis in the thyroid. Proc Natl Acad Sci USA (2007) 104:5026–31. doi: 10.1073/pnas.0606697104 PMC1829258 17360402

[117] Luther SA Ansel KM Cyster JG. Overlapping roles of cxcl13, interleukin 7 receptor alpha, and ccr7 ligands in lymph node development. J Exp Med (2003) 197:1191–8. doi: 10.1084/jem.20021294 PMC2193976 12732660

[118] Mebius RE. Organogenesis of lymphoid tissues. Nat Rev Immunol (2003) 3:292–303. doi: 10.1038/nri1054 12669020

[119] Martinet L Garrido I Filleron T Le Guellec S Bellard E Fournie JJ. Human solid tumors contain high endothelial venules: association with T- and B-lymphocyte infiltration and favorable prognosis in breast cancer. Cancer Res (2011) 71:5678–87. doi: 10.1158/0008-5472.CAN-11-0431 21846823

[120] Mionnet C Sanos SL Mondor I Jorquera A Laugier JP Germain RN. High endothelial venules as traffic control points maintaining lymphocyte population homeostasis in lymph nodes. Blood (2011) 118:6115–22. doi: 10.1182/blood-2011-07-367409 PMC3234668 21937697

[121] Ruddle NH. High endothelial venules and lymphatic vessels in tertiary lymphoid organs: characteristics, functions, and regulation. Front Immunol (2016) 7:491. doi: 10.3389/fimmu.2016.00491 27881983 PMC5101196

[122] Vella G Guelfi S Bergers G. High endothelial venules: A vascular perspective on tertiary lymphoid structures in cancer. Front Immunol (2021) 12:736670. doi: 10.3389/fimmu.2021.736670 34484246 PMC8416033

[123] Choe K Moon J Lee SY Song E Back JH Song JH. Stepwise transmigration of T- and B cells through a perivascular channel in high endothelial venules. Life Sci Alliance (2021) 4:736670–685. doi: 10.26508/lsa.202101086 PMC9715433 34187874

[124] Fridman WH Meylan M Petitprez F Sun CM Italiano A Sautes-Fridman C. B cells and tertiary lymphoid structures as determinants of tumour immune contexture and clinical outcome. Nat Rev Clin Oncol (2022) 19:441–57. doi: 10.1038/s41571-022-00619-z 35365796

[125] Goc J Germain C Vo-Bourgais TK Lupo A Klein C Knockaert S. Dendritic cells in tumor-associated tertiary lymphoid structures signal a th1 cytotoxic immune contexture and license the positive prognostic value of infiltrating cd8+ T cells. Cancer Res (2014) 74:705–15. doi: 10.1158/0008-5472.CAN-13-1342 24366885

[126] Gu-Trantien C Loi S Garaud S Equeter C Libin M de Wind A. Cd4(+) follicular helper T cell infiltration predicts breast cancer survival. J Clin Invest (2013) 123:2873–92. doi: 10.1172/JCI67428 PMC3696556 23778140

[127] Hennequin A Derangere V Boidot R Apetoh L Vincent J Orry D. Tumor infiltration by tbet+ Effector T cells and cd20+ B cells is associated with survival in gastric cancer patients. Oncoimmunology (2016) 5:e1054598. doi: 10.1080/2162402X.2015.1054598 27057426 PMC4801425

[128] Baratin M Simon L Jorquera A Ghigo C Dembele D Nowak J. T cell zone resident macrophages silently dispose of apoptotic cells in the lymph node. Immunity (2017) 47:349–62.e5. doi: 10.1016/j.immuni.2017.07.019 28801233

[129] Schumacher TN Thommen DS. Tertiary lymphoid structures in cancer. Science (2022) 375:eabf9419. doi: 10.1126/science.abf9419 34990248

[130] Di Caro G Bergomas F Grizzi F Doni A Bianchi P Malesci A. Occurrence of tertiary lymphoid tissue is associated with T-cell infiltration and predicts better prognosis in early-stage colorectal cancers. Clin Cancer Res (2014) 20:2147–58. doi: 10.1158/1078-0432.CCR-13-2590 24523438

[131] Germain C Gnjatic S Tamzalit F Knockaert S Remark R Goc J. Presence of B cells in tertiary lymphoid structures is associated with a protective immunity in patients with lung cancer. Am J Respir Crit Care Med (2014) 189:832–44. doi: 10.1164/rccm.201309-1611OC 24484236

[132] Kroeger DR Milne K Nelson BH. Tumor-infiltrating plasma cells are associated with tertiary lymphoid structures, cytolytic T-cell responses, and superior prognosis in ovarian cancer. Clin Cancer Res (2016) 22:3005–15. doi: 10.1158/1078-0432.CCR-15-2762 26763251

[133] Ishigami E Sakakibara M Sakakibara J Masuda T Fujimoto H Hayama S. Coexistence of regulatory B cells and regulatory T cells in tumor-infiltrating lymphocyte aggregates is a prognostic factor in patients with breast cancer. Breast Cancer (2019) 26:180–9. doi: 10.1007/s12282-018-0910-4 30244409

[134] Wei X Jin Y Tian Y Zhang H Wu J Lu W. Regulatory B cells contribute to the impaired antitumor immunity in ovarian cancer patients. Tumour Biol (2016) 37:6581–8. doi: 10.1007/s13277-015-4538-0 26638169

[135] Sautes-Fridman C Petitprez F Calderaro J Fridman WH. Tertiary lymphoid structures in the era of cancer immunotherapy. Nat Rev Cancer (2019) 19:307–25. doi: 10.1038/s41568-019-0144-6 31092904

[136] Moyron-Quiroz JE Rangel-Moreno J Hartson L Kusser K Tighe MP Klonowski KD. Persistence and responsiveness of immunologic memory in the absence of secondary lymphoid organs. Immunity (2006) 25:643–54. doi: 10.1016/j.immuni.2006.08.022 17045819

[137] Pitzalis C Jones GW Bombardieri M Jones SA. Ectopic lymphoid-like structures in infection, cancer and autoimmunity. Nat Rev Immunol (2014) 14:447–62. doi: 10.1038/nri3700 24948366

[138] Curiel TJ Coukos G Zou L Alvarez X Cheng P Mottram P. Specific recruitment of regulatory T cells in ovarian carcinoma fosters immune privilege and predicts reduced survival. Nat Med (2004) 10:942–9. doi: 10.1038/nm1093 15322536

[139] Dieu-Nosjean MC Antoine M Danel C Heudes D Wislez M Poulot V. Long-term survival for patients with non-small-cell lung cancer with intratumoral lymphoid structures. J Clin Oncol (2008) 26:4410–7. doi: 10.1200/JCO.2007.15.0284 18802153

[140] Wang B Liu J Han Y Deng Y Li J Jiang Y. The presence of tertiary lymphoid structures provides new insight into the clinicopathological features and prognosis of patients with breast cancer. Front Immunol (2022) 13:868155. doi: 10.3389/fimmu.2022.868155 35664009 PMC9161084

[141] Hiraoka N Ino Y Yamazaki-Itoh R Kanai Y Kosuge T Shimada K. Intratumoral tertiary lymphoid organ is a favourable prognosticator in patients with pancreatic cancer. Br J Cancer (2015) 112:1782–90. doi: 10.1038/bjc.2015.145 PMC4647237 25942397

[142] Calderaro J Petitprez F Becht E Laurent A Hirsch TZ Rousseau B. Intra-tumoral tertiary lymphoid structures are associated with a low risk of early recurrence of hepatocellular carcinoma. J Hepatol (2019) 70:58–65. doi: 10.1016/j.jhep.2018.09.003 30213589

[143] Lin Q Tao P Wang J Ma L Jiang Q Li J. Tumor-associated tertiary lymphoid structure predicts postoperative outcomes in patients with primary gastrointestinal stromal tumors. Oncoimmunology (2020) 9:1747339. doi: 10.1080/2162402X.2020.1747339 32313726 PMC7153826

[144] Cabrita R Lauss M Sanna A Donia M Skaarup Larsen M Mitra S. Tertiary lymphoid structures improve immunotherapy and survival in melanoma. Nature (2020) 577:561–5. doi: 10.1038/s41586-019-1914-8 31942071

[145] Asam S Nayar S Gardner D Barone F. Stromal cells in tertiary lymphoid structures: architects of autoimmunity. Immunol Rev (2021) 302:184–95. doi: 10.1111/imr.12987 34060101

[146] McMullen TP Lai R Dabbagh L Wallace TM de Gara CJ. Survival in rectal cancer is predicted by T cell infiltration of tumour-associated lymphoid nodules. Clin Exp Immunol (2010) 161:81–8. doi: 10.1111/j.1365-2249.2010.04147.x PMC2940152 20408858

[147] Bates GJ Fox SB Han C Leek RD Garcia JF Harris AL. Quantification of regulatory T cells enables the identification of high-risk breast cancer patients and those at risk of late relapse. J Clin Oncol (2006) 24:5373–80. doi: 10.1200/JCO.2006.05.9584 17135638

[148] Gao Q Qiu SJ Fan J Zhou J Wang XY Xiao YS. Intratumoral balance of regulatory and cytotoxic T cells is associated with prognosis of hepatocellular carcinoma after resection. J Clin Oncol (2007) 25:2586–93. doi: 10.1200/JCO.2006.09.4565 17577038

[149] Joshi NS Akama-Garren EH Lu Y Lee DY Chang GP Li A. Regulatory T cells in tumor-associated tertiary lymphoid structures suppress anti-tumor T cell responses. Immunity (2015) 43:579–90. doi: 10.1016/j.immuni.2015.08.006 PMC4826619 26341400

[150] Fridman WH Meylan M Pupier G Calvez A Hernandez I Sautes-Fridman C. Tertiary lymphoid structures and B cells: an intratumoral immunity cycle. Immunity (2023) 56:2254–69. doi: 10.1016/j.immuni.2023.08.009 37699391

[151] Helmink BA Reddy SM Gao J Zhang S Basar R Thakur R. B cells and tertiary lymphoid structures promote immunotherapy response. Nature (2020) 577:549–55. doi: 10.1038/s41586-019-1922-8 PMC8762581 31942075

[152] Petitprez F de Reynies A Keung EZ Chen TW Sun CM Calderaro J. B cells are associated with survival and immunotherapy response in sarcoma. Nature (2020) 577:556–60. doi: 10.1038/s41586-019-1906-8 31942077

[153] Vanhersecke L Brunet M Guegan JP Rey C Bougouin A Cousin S. Mature tertiary lymphoid structures predict immune checkpoint inhibitor efficacy in solid tumors independently of pd-L1 expression. Nat Cancer (2021) 2:794–802. doi: 10.1038/s43018-021-00232-6 35118423 PMC8809887

[154] Cottrell TR Thompson ED Forde PM Stein JE Duffield AS Anagnostou V. Pathologic features of response to neoadjuvant anti-pd-1 in resected non-small-cell lung carcinoma: A proposal for quantitative immune-related pathologic response criteria (Irprc). Ann Oncol (2018) 29:1853–60. doi: 10.1093/annonc/mdy218 PMC6096736 29982279

[155] Thommen DS Koelzer VH Herzig P Roller A Trefny M Dimeloe S. A transcriptionally and functionally distinct pd-1(+) cd8(+) T cell pool with predictive potential in non-small-cell lung cancer treated with pd-1 blockade. Nat Med (2018) 24:994–1004. doi: 10.1038/s41591-018-0057-z 29892065 PMC6110381

[156] Meylan M Petitprez F Becht E Bougouin A Pupier G Calvez A. Tertiary lymphoid structures generate and propagate anti-tumor antibody-producing plasma cells in renal cell cancer. Immunity (2022) 55:527–41 e5. doi: 10.1016/j.immuni.2022.02.001 35231421

[157] Gao J Navai N Alhalabi O Siefker-Radtke A Campbell MT Tidwell RS. Neoadjuvant pd-L1 plus ctla-4 blockade in patients with cisplatin-ineligible operable high-risk urothelial carcinoma. Nat Med (2020) 26:1845–51. doi: 10.1038/s41591-020-1086-y PMC9768836 33046869

[158] Lee-Chang C Bodogai M Martin-Montalvo A Wejksza K Sanghvi M Moaddel R. Inhibition of breast cancer metastasis by resveratrol-mediated inactivation of tumor-evoked regulatory B cells. J Immunol (2013) 191:4141–51. doi: 10.4049/jimmunol.1300606 PMC3795852 24043896

[159] Roya N Fatemeh T Faramarz MA Milad SG Mohammad-Javad S Najmeh SV. Frequency of il-10+Cd19+ B cells in patients with prostate cancer compared to patients with benign prostatic hyperplasia. Afr Health Sci (2020) 20:1264–72. doi: 10.4314/ahs.v20i3.31 PMC7751534 33402974

[160] Wang WW Yuan XL Chen H Xie GH Ma YH Zheng YX. Cd19+Cd24hicd38hibregs involved in downregulate helper T cells and upregulate regulatory T cells in gastric cancer. Oncotarget (2015) 6:33486–99. doi: 10.18632/oncotarget.5588 PMC4741780 26378021

[161] Allen E Jabouille A Rivera LB Lodewijckx I Missiaen R Steri V. Combined antiangiogenic and anti-pd-L1 therapy stimulates tumor immunity through hev formation. Sci Transl Med (2017) 9:eaak9679–707. doi: 10.1126/scitranslmed.aak9679 28404866 PMC5554432

[162] Lutz ER Wu AA Bigelow E Sharma R Mo G Soares K. Immunotherapy converts nonimmunogenic pancreatic tumors into immunogenic foci of immune regulation. Cancer Immunol Res (2014) 2:616–31. doi: 10.1158/2326-6066.CIR-14-0027 PMC4082460 24942756

[163] Maldonado L Teague JE Morrow MP Jotova I Wu TC Wang C. Intramuscular therapeutic vaccination targeting hpv16 induces T cell responses that localize in mucosal lesions. Sci Transl Med (2014) 6:221ra13. doi: 10.1126/scitranslmed.3007323 PMC4086631 24477000

[164] Karkkainen MJ Haiko P Sainio K Partanen J Taipale J Petrova TV. Vascular endothelial growth factor C is required for sprouting of the first lymphatic vessels from embryonic veins. Nat Immunol (2004) 5:74–80. doi: 10.1038/ni1013 14634646

[165] Hsu MC Pan MR Hung WC. Two birds, one stone: double hits on tumor growth and lymphangiogenesis by targeting vascular endothelial growth factor receptor 3. Cells (2019) 8:270–90. doi: 10.3390/cells8030270 PMC6468620 30901976

[166] Decio A Taraboletti G Patton V Alzani R Perego P Fruscio R. Vascular endothelial growth factor C promotes ovarian carcinoma progression through paracrine and autocrine mechanisms. Am J Pathol (2014) 184:1050–61. doi: 10.1016/j.ajpath.2013.12.030 24508126

[167] Goussia A Simou N Zagouri F Manousou K Lazaridis G Gogas H. Associations of angiogenesis-related proteins with specific prognostic factors, breast cancer subtypes and survival outcome in early-stage breast cancer patients. A hellenic cooperative oncology group (Hecog) trial. . PloS One (2018) 13:e0200302. doi: 10.1371/journal.pone.0200302 30063723 PMC6067711

[168] Wang J Taylor A Showeil R Trivedi P Horimoto Y Bagwan I. Expression profiling and significance of vegf-a, vegfr2, vegfr3 and related proteins in endometrial carcinoma. Cytokine (2014) 68:94–100. doi: 10.1016/j.cyto.2014.04.005 24845798

[169] Yang ZS Xu YF Huang FF Ding GF. Associations of nm23h1, vegf-C, and vegf-3 receptor in human prostate cancer. Molecules (2014) 19:6851–62. doi: 10.3390/molecules19056851 PMC6271091 24858271

[170] Zhu G Huang Q Zheng W Huang Y Hua J Yang S. Lps upregulated vegfr-3 expression promote migration and invasion in colorectal cancer via a mechanism of increased nf-kappab binding to the promoter of vegfr-3. Cell Physiol Biochem (2016) 39:1665–78. doi: 10.1159/000447868 27639612

[171] Lund AW Duraes FV Hirosue S Raghavan VR Nembrini C Thomas SN. Vegf-C promotes immune tolerance in B16 melanomas and cross-presentation of tumor antigen by lymph node lymphatics. Cell Rep (2012) 1:191–9. doi: 10.1016/j.celrep.2012.01.005 22832193

[172] Lee JY Park S Kim DC Yoon JH Shin SH Min WS. A vegfr-3 antagonist increases ifn-gamma expression on low functioning nk cells in acute myeloid leukemia. J Clin Immunol (2013) 33:826–37. doi: 10.1007/s10875-013-9877-2 23404187

[173] Li Y Weng Y Zhong L Chong H Chen S Sun Y. Vegfr3 inhibition chemosensitizes lung adenocarcinoma A549 cells in the tumor-associated macrophage microenvironment through upregulation of P53 and pten. Oncol Rep (2017) 38:2761–73. doi: 10.3892/or.2017.5969 PMC5780029 29048623

[174] Brose MS Nutting CM Jarzab B Elisei R Siena S Bastholt L. Sorafenib in radioactive iodine-refractory, locally advanced or metastatic differentiated thyroid cancer: A randomised, double-blind, phase 3 trial. Lancet (2014) 384:319–28. doi: 10.1016/S0140-6736(14)60421-9 PMC4366116 24768112

[175] Cheng AL Kang YK Chen Z Tsao CJ Qin S Kim JS. Efficacy and safety of sorafenib in patients in the asia-pacific region with advanced hepatocellular carcinoma: A phase iii randomised, double-blind, placebo-controlled trial. Lancet Oncol (2009) 10:25–34. doi: 10.1016/S1470-2045(08)70285-7 19095497

[176] Demetri GD van Oosterom AT Garrett CR Blackstein ME Shah MH Verweij J. Efficacy and safety of sunitinib in patients with advanced gastrointestinal stromal tumour after failure of imatinib: A randomised controlled trial. Lancet (2006) 368:1329–38. doi: 10.1016/S0140-6736(06)69446-4 17046465

[177] Escudier B Eisen T Stadler WM Szczylik C Oudard S Siebels M. Sorafenib in advanced clear-cell renal-cell carcinoma. N Engl J Med (2007) 356:125–34. doi: 10.1056/NEJMoa060655 17215530

[178] Haas NB Manola J Uzzo RG Flaherty KT Wood CG Kane C. Adjuvant sunitinib or sorafenib for high-risk, non-metastatic renal-cell carcinoma (Ecog-acrin E2805): A double-blind, placebo-controlled, randomised, phase 3 trial. Lancet (2016) 387:2008–16. doi: 10.1016/S0140-6736(16)00559-6 PMC4878938 26969090

[179] Llovet JM Ricci S Mazzaferro V Hilgard P Gane E Blanc JF. Sorafenib in advanced hepatocellular carcinoma. N Engl J Med (2008) 359:378–90. doi: 10.1056/NEJMoa0708857 18650514

[180] Motzer RJ Haas NB Donskov F Gross-Goupil M Varlamov S Kopyltsov E. Randomized phase iii trial of adjuvant pazopanib versus placebo after nephrectomy in patients with localized or locally advanced renal cell carcinoma. J Clin Oncol (2017) 35:3916–23. doi: 10.1200/JCO.2017.73.5324 PMC6018511 28902533

[181] Motzer RJ Hutson TE Tomczak P Michaelson MD Bukowski RM Rixe O. Sunitinib versus interferon alfa in metastatic renal-cell carcinoma. N Engl J Med (2007) 356:115–24. doi: 10.1056/NEJMoa065044 17215529

[182] Ravaud A Motzer RJ Pandha HS George DJ Pantuck AJ Patel A. Adjuvant sunitinib in high-risk renal-cell carcinoma after nephrectomy. N Engl J Med (2016) 375:2246–54. doi: 10.1056/NEJMoa1611406 27718781

[183] Sleijfer S Ray-Coquard I Papai Z Le Cesne A Scurr M Schoffski P. Pazopanib, a multikinase angiogenesis inhibitor, in patients with relapsed or refractory advanced soft tissue sarcoma: A phase ii study from the european organisation for research and treatment of cancer-soft tissue and bone sarcoma group (Eortc study 62043). J Clin Oncol (2009) 27:3126–32. doi: 10.1200/JCO.2008.21.3223 19451427

[184] Sternberg CN Davis ID Mardiak J Szczylik C Lee E Wagstaff J. Pazopanib in locally advanced or metastatic renal cell carcinoma: results of a randomized phase iii trial. J Clin Oncol (2010) 28:1061–8. doi: 10.1200/JCO.2009.23.9764 20100962

[185] Yoshimatsu Y Watabe T. Emerging roles of inflammation-mediated endothelial-mesenchymal transition in health and disease. Inflammation Regener (2022) 42:9. doi: 10.1186/s41232-021-00186-3 PMC8818500 35130955

[186] Piera-Velazquez S Jimenez SA. Endothelial to mesenchymal transition: role in physiology and in the pathogenesis of human diseases. Physiol Rev (2019) 99:1281–324. doi: 10.1152/physrev.00021.2018 PMC6734087 30864875

[187] Arciniegas E Sutton AB Allen TD Schor AM. Transforming growth factor beta 1 promotes the differentiation of endothelial cells into smooth muscle-like cells in vitro. J Cell Sci (1992) 103:521–9. doi: 10.1242/jcs.103.2.521 1478952

[188] Goumans MJ van Zonneveld AJ ten Dijke P. Transforming growth factor beta-induced endothelial-to-mesenchymal transition: A switch to cardiac fibrosis? Trends Cardiovasc Med (2008) 18:293–8. doi: 10.1016/j.tcm.2009.01.001 19345316

[189] Mihira H Suzuki HI Akatsu Y Yoshimatsu Y Igarashi T Miyazono K. Tgf-beta-induced mesenchymal transition of ms-1 endothelial cells requires smad-dependent cooperative activation of rho signals and mrtf-A. J Biochem (2012) 151:145–56. doi: 10.1093/jb/mvr121 21984612

[190] van Meeteren LA ten Dijke P. Regulation of endothelial cell plasticity by tgf-beta. Cell Tissue Res (2012) 347:177–86. doi: 10.1007/s00441-011-1222-6 PMC3250609 21866313

[191] Xiao L Dudley AC. Fine-tuning vascular fate during endothelial-mesenchymal transition. J Pathol (2017) 241:25–35. doi: 10.1002/path.4814 27701751 PMC5164846

[192] Xiong J Kawagishi H Yan Y Liu J Wells QS Edmunds LR. A metabolic basis for endothelial-to-mesenchymal transition. Mol Cell (2018) 69:689–98 e7. doi: 10.1016/j.molcel.2018.01.010 29429925 PMC5816688

[193] Fan CS Chen WS Chen LL Chen CC Hsu YT Chua KV. Osteopontin-integrin engagement induces hif-1alpha-tcf12-mediated endothelial-mesenchymal transition to exacerbate colorectal cancer. Oncotarget (2018) 9:4998–5015. doi: 10.18632/oncotarget.23578 29435158 PMC5797029

[194] Fan CS Chen LL Hsu TA Chen CC Chua KV Li CP. Endothelial-mesenchymal transition harnesses hsp90alpha-secreting M2-macrophages to exacerbate pancreatic ductal adenocarcinoma. J Hematol Oncol (2019) 12:138. doi: 10.1186/s13045-019-0826-2 31847880 PMC6918594

[195] Choi SH Kim AR Nam JK Kim JM Kim JY Seo HR. Tumour-vasculature development via endothelial-to-mesenchymal transition after radiotherapy controls cd44v6(+) cancer cell and macrophage polarization. Nat Commun (2018) 9:5108. doi: 10.1038/s41467-018-07470-w 30504836 PMC6269447

[196] Huang M Liu T Ma P Mitteer RA Jr. Zhang Z Kim HJ. C-met-mediated endothelial plasticity drives aberrant vascularization and chemoresistance in glioblastoma. J Clin Invest (2016) 126:1801–14. doi: 10.1172/JCI84876 PMC4855929 27043280

[197] Huang M Zhang D Wu JY Xing K Yeo E Li C. Wnt-mediated endothelial transformation into mesenchymal stem cell-like cells induces chemoresistance in glioblastoma. Sci Transl Med (2020) 12:eaay7522–551. doi: 10.1126/scitranslmed.aay7522 32102932 PMC7261487

[198] Nie L Lyros O Medda R Jovanovic N Schmidt JL Otterson MF. Endothelial-mesenchymal transition in normal human esophageal endothelial cells cocultured with esophageal adenocarcinoma cells: role of il-1beta and tgf-beta2. Am J Physiol Cell Physiol (2014) 307:C859–77. doi: 10.1152/ajpcell.00081.2014 PMC4216936 25163519

[199] Wang SH Chang JS Hsiao JR Yen YC Jiang SS Liu SH. Tumour cell-derived wnt5b modulates in vitro lymphangiogenesis via induction of partial endothelial-mesenchymal transition of lymphatic endothelial cells. Oncogene (2017) 36:1503–15. doi: 10.1038/onc.2016.317 27593938

[200] Wilkus-Adamczyk K Brodaczewska K Majewska A Kieda C. Microenvironment commits breast tumor ecs to dedifferentiation by micro-rna-200-B-3p regulation and extracellular matrix remodeling. Front Cell Dev Biol (2023) 11:1125077. doi: 10.3389/fcell.2023.1125077 37261072 PMC10229062

[201] Zeisberg EM Potenta S Xie L Zeisberg M Kalluri R. Discovery of endothelial to mesenchymal transition as a source for carcinoma-associated fibroblasts. Cancer Res (2007) 67:10123–8. doi: 10.1158/0008-5472.CAN-07-3127 17974953

[202] Choi KJ Nam JK Kim JH Choi SH Lee YJ. Endothelial-to-mesenchymal transition in anticancer therapy and normal tissue damage. Exp Mol Med (2020) 52:781–92. doi: 10.1038/s12276-020-0439-4 PMC7272420 32467609

[203] Platel V Corre I Clere N. Endothelial-to-mesenchymal transition (Endomt): roles in tumorigenesis, metastatic extravasation and therapy resistance. J Oncol (2019) 2019:8361945. doi: 10.1155/2019/8361945 31467544 PMC6701373

[204] Maishi N Annan DA Kikuchi H Hida Y Hida K. Tumor endothelial heterogeneity in cancer progression. Cancers (Basel) (2019) 11:1511–27. doi: 10.3390/cancers11101511 PMC6826555 31600937

[205] Rogava M Braun AD van der Sluis TC Shridhar N Tuting T Gaffal E. Tumor cell intrinsic toll-like receptor 4 signaling promotes melanoma progression and metastatic dissemination. Int J Cancer (2022) 150:142–51. doi: 10.1002/ijc.33804 34528710

[206] Wu FH Yuan Y Li D Lei Z Song CW Liu YY. Endothelial cell-expressed tim-3 facilitates metastasis of melanoma cells by activating the nf-kappab pathway. Oncol Rep (2010) 24:693–9. 20664975

[207] Cong Y Cui Y Zhu S Cao J Zou H Martin TA. Tim-3 promotes cell aggressiveness and paclitaxel resistance through nf-kappab/stat3 signalling pathway in breast cancer cells. Chin J Cancer Res (2020) 32:564–79. doi: 10.21147/j.issn.1000-9604.2020.05.02 PMC7666787 33223752

[208] Cong Y Wang X Wang S Qiao G Li Y Cao J. Tim-3 promotes tube formation and decreases tight junction formation in vascular endothelial cells. Biosci Rep (2020) 40:BSR20202130–43. doi: 10.1042/BSR20202130 33015716 PMC7560514

[209] Wieland E Rodriguez-Vita J Liebler SS Mogler C Moll I Herberich SE. Endothelial notch1 activity facilitates metastasis. Cancer Cell (2017) 31:355–67. doi: 10.1016/j.ccell.2017.01.007 28238683

[210] Brantley-Sieders DM Dunaway CM Rao M Short S Hwang Y Gao Y. Angiocrine factors modulate tumor proliferation and motility through epha2 repression of slit2 tumor suppressor function in endothelium. Cancer Res (2011) 71:976–87. doi: 10.1158/0008-5472.CAN-10-3396 PMC3032824 21148069

[211] Liu Y Colby JK Zuo X Jaoude J Wei D Shureiqi I. The role of ppar-delta in metabolism, inflammation, and cancer: many characters of a critical transcription factor. Int J Mol Sci (2018) 19:3339–53. doi: 10.3390/ijms19113339 PMC6275063 30373124

[212] Piqueras L Reynolds AR Hodivala-Dilke KM Alfranca A Redondo JM Hatae T. Activation of pparbeta/delta induces endothelial cell proliferation and angiogenesis. Arterioscler Thromb Vasc Biol (2007) 27:63–9. doi: 10.1161/01.ATV.0000250972.83623.61 17068288

[213] Abdollahi A Schwager C Kleeff J Esposito I Domhan S Peschke P. Transcriptional network governing the angiogenic switch in human pancreatic cancer. Proc Natl Acad Sci USA (2007) 104:12890–5. doi: 10.1073/pnas.0705505104 PMC1931565 17652168

[214] Du S Wagner N Wagner KD. The emerging role of ppar beta/delta in tumor angiogenesis. PPAR Res (2020) 2020:3608315. doi: 10.1155/2020/3608315 32855630 PMC7443046

[215] La Paglia L Listi A Caruso S Amodeo V Passiglia F Bazan V. Potential role of angptl4 in the cross talk between metabolism and cancer through ppar signaling pathway. PPAR Res (2017) 2017:8187235. doi: 10.1155/2017/8187235 28182091 PMC5274667

[216] Leone P Solimando AG Prete M Malerba E Susca N Derakhshani A. Unraveling the role of peroxisome proliferator-activated receptor beta/delta (Ppar beta/delta) in angiogenesis associated with multiple myeloma. Cells (2023) 12:1011–28. doi: 10.3390/cells12071011 PMC10093382 37048084

[217] Yadav A Kumar B Yu JG Old M Teknos TN Kumar P. Tumor-associated endothelial cells promote tumor metastasis by chaperoning circulating tumor cells and protecting them from anoikis. PloS One (2015) 10:e0141602. doi: 10.1371/journal.pone.0141602 26509633 PMC4624958

[218] Kontos F Michelakos T Kurokawa T Sadagopan A Schwab JH Ferrone CR. B7-H3: an attractive target for antibody-based immunotherapy. Clin Cancer Res (2021) 27:1227–35. doi: 10.1158/1078-0432.CCR-20-2584 PMC7925343 33051306

[219] Seaman S Stevens J Yang MY Logsdon D Graff-Cherry C St Croix B. Genes that distinguish physiological and pathological angiogenesis. Cancer Cell (2007) 11:539–54. doi: 10.1016/j.ccr.2007.04.017 PMC2039723 17560335

[220] Wang R Ma Y Zhan S Zhang G Cao L Zhang X. B7-H3 promotes colorectal cancer angiogenesis through activating the nf-kappab pathway to induce vegfa expression. Cell Death Dis (2020) 11:55. doi: 10.1038/s41419-020-2252-3 31974361 PMC6978425

[221] Zang X Sullivan PS Soslow RA Waitz R Reuter VE Wilton A. Tumor associated endothelial expression of B7-H3 predicts survival in ovarian carcinomas. Mod Pathol (2010) 23:1104–12. doi: 10.1038/modpathol.2010.95 PMC2976590 20495537

[222] Crispen PL Sheinin Y Roth TJ Lohse CM Kuntz SM Frigola X. Tumor cell and tumor vasculature expression of B7-H3 predict survival in clear cell renal cell carcinoma. Clin Cancer Res (2008) 14:5150–7. doi: 10.1158/1078-0432.CCR-08-0536 PMC2789387 18694993

[223] Aung PP Parra ER Barua S Sui D Ning J Mino B. B7-H3 expression in merkel cell carcinoma-associated endothelial cells correlates with locally aggressive primary tumor features and increased vascular density. Clin Cancer Res (2019) 25:3455–67. doi: 10.1158/1078-0432.CCR-18-2355 PMC8211110 30808776

[224] Palazon A Teijeira A Martinez-Forero I Hervas-Stubbs S Roncal C Penuelas I. Agonist anti-cd137 mab act on tumor endothelial cells to enhance recruitment of activated T lymphocytes. Cancer Res (2011) 71:801–11. doi: 10.1158/0008-5472.CAN-10-1733 21266358

[225] Maishi N Ohba Y Akiyama K Ohga N Hamada J Nagao-Kitamoto H. Tumour endothelial cells in high metastatic tumours promote metastasis via epigenetic dysregulation of biglycan. Sci Rep (2016) 6:28039. doi: 10.1038/srep28039 27295191 PMC4904795

[226] Liu FT Rabinovich GA. Galectins as modulators of tumour progression. Nat Rev Cancer (2005) 5:29–41. doi: 10.1038/nrc1527 15630413

[227] Inufusa H Nakamura M Adachi T Aga M Kurimoto M Nakatani Y. Role of galectin-3 in adenocarcinoma liver metastasis. Int J Oncol (2001) 19:913–9. doi: 10.3892/ijo.19.5.913 11604988

[228] Song YK Billiar TR Lee YJ. Role of galectin-3 in breast cancer metastasis: involvement of nitric oxide. Am J Pathol (2002) 160:1069–75. doi: 10.1016/S0002-9440(10)64927-9 PMC1867157 11891203

[229] Croci DO Cerliani JP Dalotto-Moreno T Mendez-Huergo SP Mascanfroni ID Dergan-Dylon S. Glycosylation-dependent lectin-receptor interactions preserve angiogenesis in anti-vegf refractory tumors. Cell (2014) 156:744–58. doi: 10.1016/j.cell.2014.01.043 24529377

[230] Delgado VM Nugnes LG Colombo LL Troncoso MF Fernandez MM Malchiodi EL. Modulation of endothelial cell migration and angiogenesis: A novel function for the "Tandem-repeat" Lectin galectin-8. FASEB J (2011) 25:242–54. doi: 10.1096/fj.09-144907 20876211

[231] He J Baum LG. Endothelial cell expression of galectin-1 induced by prostate cancer cells inhibits T-cell transendothelial migration. Lab Invest (2006) 86:578–90. doi: 10.1038/labinvest.3700420 16607379

[232] Hsieh SH Ying NW Wu MH Chiang WF Hsu CL Wong TY. Galectin-1, a novel ligand of neuropilin-1, activates vegfr-2 signaling and modulates the migration of vascular endothelial cells. Oncogene (2008) 27:3746–53. doi: 10.1038/sj.onc.1211029 18223683

[233] Nangia-Makker P Honjo Y Sarvis R Akahani S Hogan V Pienta KJ. Galectin-3 induces endothelial cell morphogenesis and angiogenesis. Am J Pathol (2000) 156:899–909. doi: 10.1016/S0002-9440(10)64959-0 10702407 PMC1876842

[234] Thijssen VL Postel R Brandwijk RJ Dings RP Nesmelova I Satijn S. Galectin-1 is essential in tumor angiogenesis and is a target for antiangiogenesis therapy. Proc Natl Acad Sci USA (2006) 103:15975–80. doi: 10.1073/pnas.0603883103 PMC1635112 17043243

[235] Markowska AI Liu FT Panjwani N. Galectin-3 is an important mediator of vegf- and bfgf-mediated angiogenic response. J Exp Med (2010) 207:1981–93. doi: 10.1084/jem.20090121 PMC2931172 20713592

[236] Markowska AI Jefferies KC Panjwani N. Galectin-3 protein modulates cell surface expression and activation of vascular endothelial growth factor receptor 2 in human endothelial cells. J Biol Chem (2011) 286:29913–21. doi: 10.1074/jbc.M111.226423 PMC3191032 21715322

[237] White NM Masui O Newsted D Scorilas A Romaschin AD Bjarnason GA. Galectin-1 has potential prognostic significance and is implicated in clear cell renal cell carcinoma progression through the hif/mtor signaling axis. Br J Cancer (2014) 110:1250–9. doi: 10.1038/bjc.2013.828 PMC3950857 24496460

[238] Zhao XY Zhao KW Jiang Y Zhao M Chen GQ. Synergistic induction of galectin-1 by ccaat/enhancer binding protein alpha and hypoxia-inducible factor 1alpha and its role in differentiation of acute myeloid leukemic cells. J Biol Chem (2011) 286:36808–19. doi: 10.1074/jbc.M111.247262 PMC3196150 21880716

[239] Laderach DJ Gentilini LD Giribaldi L Delgado VC Nugnes L Croci DO. A unique galectin signature in human prostate cancer progression suggests galectin-1 as a key target for treatment of advanced disease. Cancer Res (2013) 73:86–96. doi: 10.1158/0008-5472.CAN-12-1260 23108139

[240] Femel J van Hooren L Herre M Cedervall J Saupe F Huijbers EJM. Vaccination against galectin-1 promotes cytotoxic T-cell infiltration in melanoma and reduces tumor burden. Cancer Immunol Immunother (2022) 71:2029–40. doi: 10.1007/s00262-021-03139-4 PMC9293851 35018481

[241] Chen WS Cao Z Sugaya S Lopez MJ Sendra VG Laver N. Pathological lymphangiogenesis is modulated by galectin-8-dependent crosstalk between podoplanin and integrin-associated vegfr-3. Nat Commun (2016) 7:11302. doi: 10.1038/ncomms11302 27066737 PMC4832077

[242] Haibe Y Kreidieh M El Hajj H Khalifeh I Mukherji D Temraz S. Resistance mechanisms to anti-angiogenic therapies in cancer. Front Oncol (2020) 10:221. doi: 10.3389/fonc.2020.00221 32175278 PMC7056882

[243] Cao Z Ding BS Guo P Lee SB Butler JM Casey SC. Angiocrine factors deployed by tumor vascular niche induce B cell lymphoma invasiveness and chemoresistance. Cancer Cell (2014) 25:350–65. doi: 10.1016/j.ccr.2014.02.005 PMC4017921 24651014

[244] Huinen ZR Huijbers EJM van Beijnum JR Nowak-Sliwinska P Griffioen AW. Anti-angiogenic agents - overcoming tumour endothelial cell anergy and improving immunotherapy outcomes. Nat Rev Clin Oncol (2021) 18:527–40. doi: 10.1038/s41571-021-00496-y 33833434

[245] Jain RK. Normalizing tumor vasculature with anti-angiogenic therapy: A new paradigm for combination therapy. Nat Med (2001) 7:987–9. doi: 10.1038/nm0901-987 11533692

[246] Jain RK. Antiangiogenesis strategies revisited: from starving tumors to alleviating hypoxia. Cancer Cell (2014) 26:605–22. doi: 10.1016/j.ccell.2014.10.006 PMC4269830 25517747

[247] Willett CG Boucher Y di Tomaso E Duda DG Munn LL Tong RT. Direct evidence that the vegf-specific antibody bevacizumab has antivascular effects in human rectal cancer. Nat Med (2004) 10:145–7. doi: 10.1038/nm988 PMC2693485 14745444

[248] Batchelor TT Sorensen AG di Tomaso E Zhang WT Duda DG Cohen KS. Azd2171, a pan-vegf receptor tyrosine kinase inhibitor, normalizes tumor vasculature and alleviates edema in glioblastoma patients. Cancer Cell (2007) 11:83–95. doi: 10.1016/j.ccr.2006.11.021 17222792 PMC2748664

[249] Gerstner ER Ye X Duda DG Levine MA Mikkelsen T Kaley TJ. A phase I study of cediranib in combination with cilengitide in patients with recurrent glioblastoma. Neuro Oncol (2015) 17:1386–92. doi: 10.1093/neuonc/nov085 PMC4578584 26008604

[250] Sorensen AG Batchelor TT Zhang WT Chen PJ Yeo P Wang M. A "Vascular normalization index" as potential mechanistic biomarker to predict survival after a single dose of cediranib in recurrent glioblastoma patients. Cancer Res (2009) 69:5296–300. doi: 10.1158/0008-5472.CAN-09-0814 PMC2824172 19549889

[251] Sorensen AG Emblem KE Polaskova P Jennings D Kim H Ancukiewicz M. Increased survival of glioblastoma patients who respond to antiangiogenic therapy with elevated blood perfusion. Cancer Res (2012) 72:402–7. doi: 10.1158/0008-5472.CAN-11-2464 PMC3261301 22127927

[252] Huang Y Yuan J Righi E Kamoun WS Ancukiewicz M Nezivar J. Vascular normalizing doses of antiangiogenic treatment reprogram the immunosuppressive tumor microenvironment and enhance immunotherapy. Proc Natl Acad Sci USA (2012) 109:17561–6. doi: 10.1073/pnas.1215397109 PMC3491458 23045683

[253] Chinnasamy D Yu Z Theoret MR Zhao Y Shrimali RK Morgan RA. Gene therapy using genetically modified lymphocytes targeting vegfr-2 inhibits the growth of vascularized syngenic tumors in mice. J Clin Invest (2010) 120:3953–68. doi: 10.1172/JCI43490 PMC2964987 20978347

[254] Tian L Goldstein A Wang H Ching Lo H Sun Kim I Welte T. Mutual regulation of tumour vessel normalization and immunostimulatory reprogramming. Nature (2017) 544:250–4. doi: 10.1038/nature21724 PMC5788037 28371798

[255] Li Q Wang Y Jia W Deng H Li G Deng W. Low-dose anti-angiogenic therapy sensitizes breast cancer to pd-1 blockade. Clin Cancer Res (2020) 26:1712–24. doi: 10.1158/1078-0432.CCR-19-2179 31848190

[256] Schmittnaegel M Rigamonti N Kadioglu E Cassara A Wyser Rmili C Kiialainen A. Dual angiopoietin-2 and vegfa inhibition elicits antitumor immunity that is enhanced by pd-1 checkpoint blockade. Sci Transl Med (2017) 9:eaak9670–84. doi: 10.1126/scitranslmed.aak9670 28404865

[257] Wu X Giobbie-Hurder A Liao X Lawrence D McDermott D Zhou J. Vegf neutralization plus ctla-4 blockade alters soluble and cellular factors associated with enhancing lymphocyte infiltration and humoral recognition in melanoma. Cancer Immunol Res (2016) 4:858–68. doi: 10.1158/2326-6066.CIR-16-0084 PMC5050160 27549123

[258] Rini BI Powles T Atkins MB Escudier B McDermott DF Suarez C. Atezolizumab plus bevacizumab versus sunitinib in patients with previously untreated metastatic renal cell carcinoma (Immotion151): A multicentre, open-label, phase 3, randomised controlled trial. Lancet (2019) 393:2404–15. doi: 10.1016/S0140-6736(19)30723-8 31079938

[259] Wallin JJ Bendell JC Funke R Sznol M Korski K Jones S. Atezolizumab in combination with bevacizumab enhances antigen-specific T-cell migration in metastatic renal cell carcinoma. Nat Commun (2016) 7:12624. doi: 10.1038/ncomms12624 27571927 PMC5013615

[260] Finn RS Qin S Ikeda M Galle PR Ducreux M Kim TY. Atezolizumab plus bevacizumab in unresectable hepatocellular carcinoma. N Engl J Med (2020) 382:1894–905. doi: 10.1056/NEJMoa1915745 32402160

[261] Socinski MA Jotte RM Cappuzzo F Orlandi F Stroyakovskiy D Nogami N. Atezolizumab for first-line treatment of metastatic nonsquamous nsclc. N Engl J Med (2018) 378:2288–301. doi: 10.1056/NEJMoa1716948 29863955

[262] Lee J Koh J Kim HK Hong S Kim K Park S. Bevacizumab plus atezolizumab after progression on atezolizumab monotherapy in pretreated patients with nsclc: an open-label, two-stage, phase 2 trial. J Thorac Oncol (2022) 17:900–8. doi: 10.1016/j.jtho.2022.04.001 35427805

[263] Cui X Jia H Xin H Zhang L Chen S Xia S. A novel bispecific antibody targeting pd-L1 and vegf with combined anti-tumor activities. Front Immunol (2021) 12:778978. doi: 10.3389/fimmu.2021.778978 34925354 PMC8678608

[264] Du YQ Zhang J Sun ML Wen Q Zheng YW Tolcher AW. Safety and efficacy of hb0025, an anti-pd-1/vegf bispecific antibody fusion protein, in patients with advanced solid tumors: preliminary results from an fih trial. J Clin Oncol (2023) 41. doi: 10.1200/JCO.2023.41.16_suppl.2589

[265] Saba NF Steuer CE Ekpenyong A McCook-Veal A Magliocca K Patel M. Pembrolizumab and cabozantinib in recurrent metastatic head and neck squamous cell carcinoma: A phase 2 trial. Nat Med (2023) 29:880–7. doi: 10.1038/s41591-023-02275-x PMC10205145 37012550

[266] Procopio G Claps M Pircher C Porcu L Sepe P Guadalupi V. A multicenter phase 2 single arm study of cabozantinib in patients with advanced or unresectable renal cell carcinoma pre-treated with one immune-checkpoint inhibitor: the breakpoint trial (Meet-uro trial 03). Tumori (2023) 109:129–37. doi: 10.1177/03008916221138881 PMC9896529 36447337

[267] Li B Jin J Guo D Tao Z Hu X. Immune checkpoint inhibitors combined with targeted therapy: the recent advances and future potentials. Cancers (Basel) (2023) 15:2858–78. doi: 10.3390/cancers15102858 PMC10216018 37345194

[268] Rossi E Bersanelli M Gelibter AJ Borsellino N Caserta C Doni L. Combination therapy in renal cell carcinoma: the best choice for every patient? Curr Oncol Rep (2021) 23:147. doi: 10.1007/s11912-021-01140-9 34748099 PMC8575734

[269] Cheng AL Qin S Ikeda M Galle PR Ducreux M Kim TY. Updated efficacy and safety data from imbrave150: atezolizumab plus bevacizumab vs. Sorafenib for unresectable hepatocellular carcinoma. J Hepatol (2022) 76:862–73. doi: 10.1016/j.jhep.2021.11.030 34902530

[270] Choueiri TK Motzer RJ Rini BI Haanen J Campbell MT Venugopal B. Updated efficacy results from the javelin renal 101 trial: first-line avelumab plus axitinib versus sunitinib in patients with advanced renal cell carcinoma. Ann Oncol (2020) 31:1030–9. doi: 10.1016/j.annonc.2020.04.010 PMC8436592 32339648

[271] Emens LA Esteva FJ Beresford M Saura C De Laurentiis M Kim SB. Trastuzumab emtansine plus atezolizumab versus trastuzumab emtansine plus placebo in previously treated, her2-positive advanced breast cancer (Kate2): A phase 2, multicentre, randomised, double-blind trial. Lancet Oncol (2020) 21:1283–95. doi: 10.1016/S1470-2045(20)30465-4 33002436

[272] Lee MS Ryoo BY Hsu CH Numata K Stein S Verret W. Atezolizumab with or without bevacizumab in unresectable hepatocellular carcinoma (Go30140): an open-label, multicentre, phase 1b study. Lancet Oncol (2020) 21:808–20. doi: 10.1016/S1470-2045(20)30156-X 32502443

[273] Socinski MA Nishio M Jotte RM Cappuzzo F Orlandi F Stroyakovskiy D. Impower150 final overall survival analyses for atezolizumab plus bevacizumab and chemotherapy in first-line metastatic nonsquamous nsclc. J Thorac Oncol (2021) 16:1909–24. doi: 10.1016/j.jtho.2021.07.009 34311108

[274] Zhu AX Abbas AR de Galarreta MR Guan Y Lu S Koeppen H. Molecular correlates of clinical response and resistance to atezolizumab in combination with bevacizumab in advanced hepatocellular carcinoma. Nat Med (2022) 28:1599–611. doi: 10.1038/s41591-022-01868-2 35739268

[275] Lu L Zhan M Li XY Zhang H Dauphars DJ Jiang J. Clinically approved combination immunotherapy: current status, limitations, and future perspective. Curr Res Immunol (2022) 3:118–27. doi: 10.1016/j.crimmu.2022.05.003 PMC9167882 35676925

[276] Chen J Siliceo SL Ni Y Nielsen HB Xu A Panagiotou G. Identification of robust and generalizable biomarkers for microbiome-based stratification in lifestyle interventions. Microbiome (2023) 11:178. doi: 10.1186/s40168-023-01604-z 37553697 PMC10408196

[277] Gao J Zhang X Jiang L Li Y Zheng Q. Tumor endothelial cell-derived extracellular vesicles contribute to tumor microenvironment remodeling. Cell Commun Signal (2022) 20:97. doi: 10.1186/s12964-022-00904-5 35752798 PMC9233793

[278] Zhang S Yang J Shen L. Extracellular vesicle-mediated regulation of tumor angiogenesis- implications for anti-angiogenesis therapy. J Cell Mol Med (2021) 25:2776–85. doi: 10.1111/jcmm.16359 PMC7957215 33586248

[279] Yang K Han W Jiang X Piffko A Bugno J Han C. Zinc cyclic di-amp nanoparticles target and suppress tumours via endothelial sting activation and tumour-associated macrophage reinvigoration. Nat Nanotechnol (2022) 17:1322–31. doi: 10.1038/s41565-022-01225-x 36302963

